# FRMD3 inhibits the growth and metastasis of breast cancer through the ubiquitination-mediated degradation of vimentin and subsequent impairment of focal adhesion

**DOI:** 10.1038/s41419-023-05552-2

**Published:** 2023-01-11

**Authors:** Wenjun Shao, Jiawei Li, Qianling Piao, Xinlei Yao, Mingyue Li, Shuyue Wang, Zhenbo Song, Ying Sun, Lihua Zheng, Guannan Wang, Lei Liu, Chunlei Yu, Yanxin Huang, Yongli Bao, Luguo Sun

**Affiliations:** 1 National Engineering Laboratory for Druggable Gene and Protein Screening, Northeast Normal University, Changchun, 130024 China; 2 NMPA Key Laboratory for Quality of Cell and Gene Therapy Medicinal Products, Northeast Normal University, Changchun, 130024 China

**Keywords:** Focal adhesion, Breast cancer

## Abstract

Recurrence and metastasis are the main causes of breast cancer (BRCA)-related death and remain a challenge for treatment. In-depth research on the molecular mechanisms underlying BRCA progression has been an important basis for developing precise biomarkers and therapy targets for early prediction and treatment of progressed BRCA. Herein, we identified FERM domain-containing protein 3 (FRMD3) as a novel potent BRCA tumor suppressor which is significantly downregulated in BRCA clinical tissue and cell lines, and low FRMD3 expression has been closely associated with progressive BRCA and shortened survival time in BRCA patients. Overexpression and knockdown experiments have revealed that FRMD3 significantly inhibits BRCA cell proliferation, migration, and invasion in vitro and suppresses BRCA xenograft growth and metastasis in vivo as well. Mechanistically, FRMD3 can interact with vimentin and ubiquitin protein ligase E3A(UBE3A) to induce the polyubiquitin-mediated proteasomal degradation of vimentin, which subsequently downregulates focal adhesion complex proteins and pro-cancerous signaling activation, thereby resulting in cytoskeletal rearrangement and defects in cell morphology and focal adhesion. Further evidence has confirmed that FRMD3-mediated vimentin degradation accounts for the anti-proliferation and anti-metastasis effects of FRMD3 on BRCA. Moreover, the N-terminal ubiquitin-like domain of FRMD3 has been identified as responsible for FRMD3-vimentin interaction through binding the head domain of vimentin and the truncated FRMD3 with the deletion of ubiquitin-like domain almost completely loses the anti-BRCA effects. Taken together, our study indicates significant potential for the use of FRMD3 as a novel prognosis biomarker and a therapeutic target of BRCA and provides an additional mechanism underlying the degradation of vimentin and BRCA progression.

## Introduction

Breast cancer (BRCA) is the most frequently diagnosed cancer and the primary cause of cancer death in females [1]. Despite advancements made in the diagnosis and treatment of breast cancer, there is still a risk of recurrence and distant metastasis, which is the main cause of breast cancer-related deaths [2]. Currently, the mechanism of BRCA metastasis is not yet fully understood, and it remains a challenge in the prevention and treatment of metastatic BRCA [3]. Therefore, in-depth research on the molecular mechanisms involved in BRCA development and progression is crucial for finding the biomarkers for the early prediction of metastatic risk, identifying therapeutic targets, and improving therapeutic strategies, all of which can reduce BRCA mortality and benefit patients.

FERM domain-containing protein 3 (FRMD3) is one member of the protein 4.1 superfamily, which is characterized by the presence of a conserved FERM domain. Members of this family serve as cytoskeletal proteins, maintaining cell shape and intergrity [4]. The conserved FERM domain is involved in binding membrane-related proteins and lipids, acting as the junction between cell membrane and cytoskeleton. FRMD3 expression has been found in multiple human tissues [5]. However, the role of FRMD3 has not been fully examined. To date, there is a limited number of studies on FRMD3, most of which reported the close association of FRMD3 gene polymorphisms with diabetic nephropathy [6]. Other studies have concerned with the role of FRMD3 in cancer. As early as 2007, FRMD3 was identified as a tumor suppressor gene in lung cancer, suggesting a potential role in the origin and progression of lung cancer [7]. Thereafter, high FRMD3 expression was reported to be related to advanced clinicopathological features in rectal cancer patients [8], and FRMD3 was shown to be the target of Has-miR-3651, a novel predictor for breast cancer recurrence [9]. However, the functional roles and the underlying mechanisms of FRMD3 in cancers including breast cancer remains largely unknown.

Vimentin, a type III intermediate filament, exists in the cytoplasm of mesenchymal cells and maintains cell structure and tissue integrity [10]. Through interacting with filamin A, the actin cross-linking protein [11], vimentin interacts with microfilaments and microtubules to maintain cytoskeletal structure [12] and mediates cell adhesion and spreading [13]. In addition, it has been demonstrated that vimentin can interact with β1-integrin to modulate cell focal adhesion and cellular signal transduction [14]. β1-integrin, which is responsible for the cell attachment to the extracellular matrix (ECM), can recruit focal adhesion kinase (FAK) upon stimulation and activation to form nascent adhesion [15, 16]. Hence, the lack of vimentin results in the shape changes of the intermediate filament cytoskeleton structures and the abnormality of processes related to cell adhesion, migration, and invasion [17, 18]. In cancer, vimentin is well known for being an important marker of epithelial-mesenchymal transition (EMT), a process critical for cancer invasion and metastasis cascades [19]. However, in addition to being a marker, vimentin plays an essential role in modulating cancer cell mobility and cancer metastasis since vimentin regulates cell adhesion and spreading [20]. Therefore, a high level of vimentin expression has been associated with increased risks of metastasis and represents poor prognosis in a variety of cancers, including breast cancer, prostate cancer, melanoma, and lung cancer [21]. The mechanisms leading to a high level of vimentin expression in cancer cells is worth extensive exploration as this could further our understanding of cancer metastasis and identify new methods to mitigate cancer metastasis.

In this study, we demonstrated that FRMD3, which is significantly downregulated in breast cancer, binds with vimentin through its ubiquitin-like domain, leading to the ubiquitination-mediated degradation of vimentin via recruiting ubiquitin ligase UBE3A in breast cancer cells. Therefore, FRMD3 acted as a tumor suppressor to inhibit breast cancer cell proliferation, migration, and invasion both in vitro and in vivo.

## Results

### Downregulation of FRMD3 was common in BRCA and indicated progression and poor survival

**Fig. 1:**
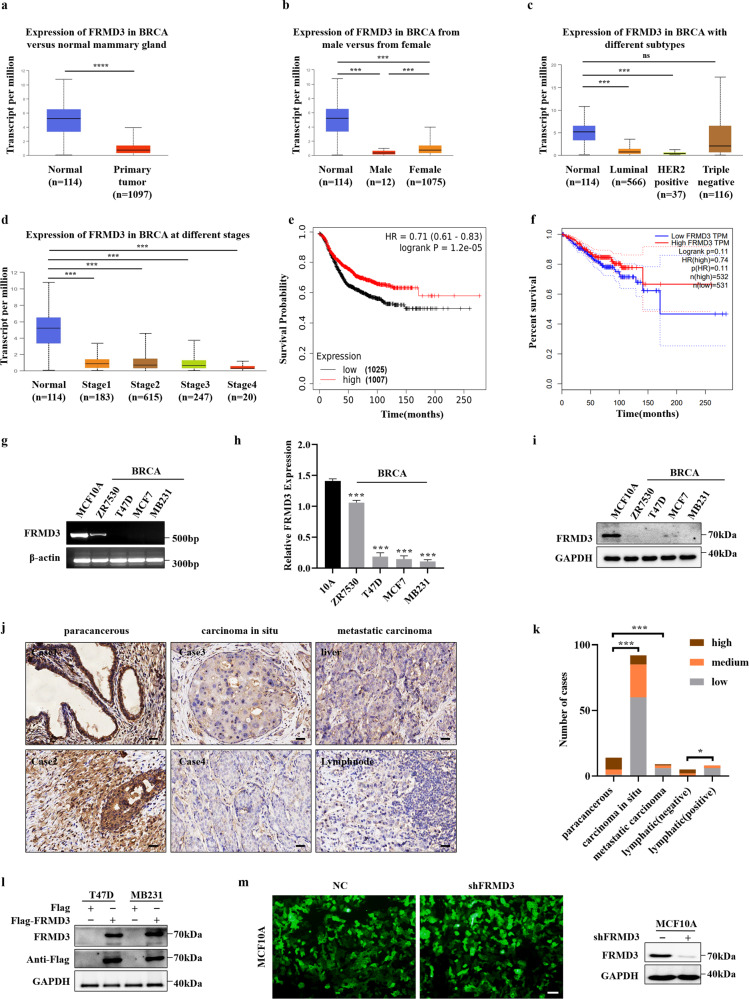
Downregulation of FRMD3 was frequently observed in BRCA samples and indicated poor survival. **a**–**d** Analysis of the mRNA levels of FRMD3 in breast cancer based on sample types (**a**), gender (**b**), major subtype (**c**), and cancer stage (**d**). These data were downloaded from TCGA database and analyzed on UALCAN. **e** Kaplan–Meier survival analysis of 2032 patients with breast cancer based on FRMD3 gene expression via Kaplan–Meier Plotter. **f** GEPIA analysis of the correlation between FRMD3 mRNA expression levels and disease-free survival of 1063 breast cancer patients. **g**–**i** RT-PCR (**g**), RT-qPCR (**h**), and Western blot (**i**) detected the expression levels of FRMD3 in MCF10A normal breast epithelial cell line and different BRCA cell lines. GAPDH and β-actin were used as internal controls. **j** Representative images of immunohistochemical (IHC) analysis of FRMD3 protein levels in the paracancerous tissues, carcinoma in situ, as well as metastatic BRCA in liver and lymph nodes on TMA slides. Scale bar, 50 μm. **k** Immunoreactivity score of FRMD3 protein levels in paracancerous tissues (**n** = 14), carcinoma in situ (*n* = 92), metastatic carcinoma (*n* = 9), lymph nodes negative for metastasis (*n* = 5), and lymph nodes positive for metastasis (positive, *n* = 8). **l** Western blot analysis of the exogenous FRMD3 protein expression in T47D and MDA-MB-231 cells with stable pcflag-FRMD3 transfection. **m** GFP fluorescent images (left) and Western blot analysis of FRMD3 levels (right) of MCF10A cells stably transfected with shFRMD3 by lentivirus. Scale bar, 100 μm. *N* = 3 biologically independent replicates. The student’s *t*-test was used to estimate the significance of difference between two groups and more than two groups were analyzed by one-way ANOVA. Data were presented as means ± s.d. ns not significant; **P < 0.01; ***P < 0.001.

FRMD3 mRNA levels in BRCA patients from TCGA database were first analyzed using UALCAN [22], which showed significantly lower mRNA levels of FRMD3 in BRCA, as compared to those in normal mammary glands (Fig. 1a). Taking gender into account, FRMD3 level was a bit lower in male BRCA than in female (Fig. 1b). Further analysis based on the main subtypes of BRCA showed that the triple-negative BRCA had the highest while the HER2^+^ BRCA had the lowest level of FRMD3 among the three subtypes of BRCA (Fig. 1c). A comparison of the FRMD3 levels in different stages of BRCA displayed a stage-dependent tendency to decrease, indicating that FRMD3 expression decreased with the progression of breast cancer (Fig. 1d). Interestingly, the patients with low FRMD3 expression had significantly shorter disease-free survival times than those with high FRMD3 expression (Fig. 1e, f). Then we detected the expression of FRMD3 in several BRCA cell lines and MCF10A normal mammary epithelial cell line by RT-PCR, qPCR, and Western blot. The results showed that FRMD3 expression significantly decreased at both mRNA and protein levels in most BRCA cell lines, as compared to MCF10A cells (Fig. 1g–i, S1a). Furthermore, FRMD3 protein was nearly indetectable in BRCA cell lines (Fig. 1i, S1a). Furthermore, the expression of FRMD3 was also detected by performing immunohistochemistry (IHC) staining of two tissue microarray (TMA) slides that contained primary and metastatic BRCA, paracancerous tissues, and lymph nodes, with or without metastatic BRCA cells as well. As shown in Fig. 1j, FRMD3 was positively stained in cytoplasm, and the positive rate and intensity of FRMD3 staining significantly decreased in BRCA tissues, as compared to those in paracancerous tissues. Further analysis of the proportions of high, medium, and low levels of FRMD3 in these tissues, as shown in Fig. 1k, indicated that a much higher proportion of low-level and a lower proportion of high-level FRMD3 was observed in primary and metastatic BRCA and lymph nodes that were positive for metastatic BRCA cells. Meanwhile, the comparison of the proportions of high, medium, and low levels of FRMD3 in different BRCA subtypes showed that there was no apparent difference (Fig. S1b). These data confirmed the significant downregulation of FRMD expression in BRCA. In addition, the FRMD3 level reversely correlated with the stages of BRCA and could serve as a diagnostic and prognostic biomarker.

### FRMD3 inhibited the proliferation of BRCA cells

**Fig. 2:**
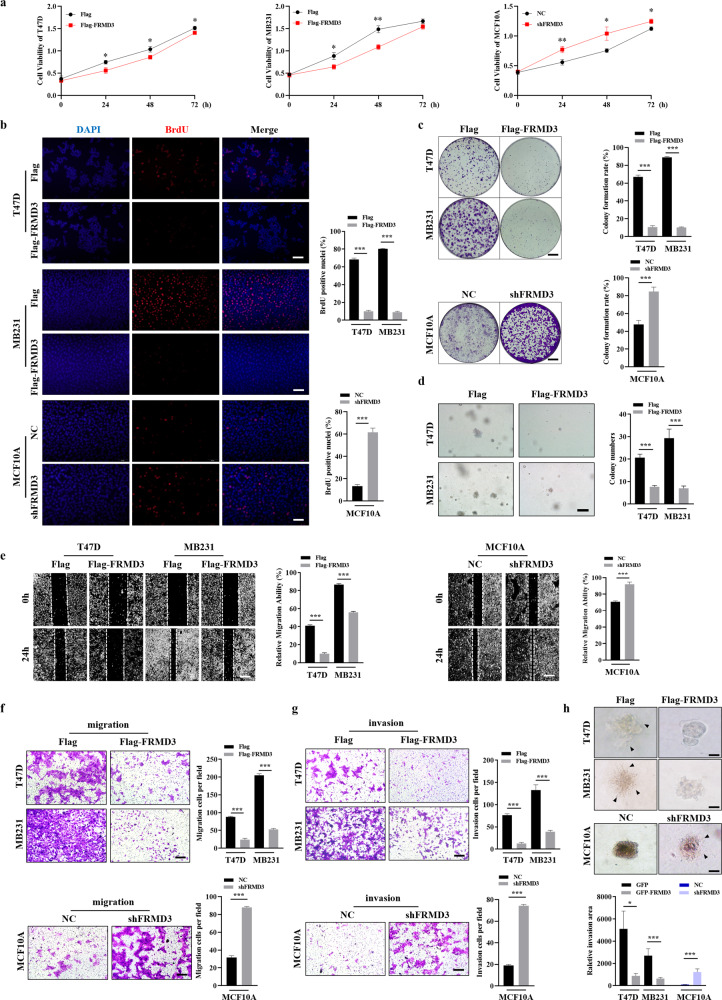
FRMD3 inhibited the proliferation, migration, and invasion of BRCA cells in vitro. **a** Cell viability of stable MCF10A with shFRMD3 or stable T47D and MDA-MB-231 cells with FRMD3 overexpression detected by MTT assay together with negative control (NC). **b** BrdU incorporation assay to evaluate cell proliferation of indicated cells by immunofluorescence staining (left) and the quantification of staining was shown on the right. Scale bar, 100 μm. **c** Representative images (left) and the quantification results (right) of colony formation assay of indicated cells. Scale bar, 200 μm. **d** Representative images of soft agar assay for colony formation of T47D and MDA-MB-231 cells with FRMD3 overexpression (left) and quantification of sphere numbers (right). Scale bar, 100 μm. **e** Scratch wound-healing assay in MCF10 cells with FRMD3 knockdown and T47D, MDA-MB-231 cells with FRMD3 overexpression. Scale bar, 200 μm. **f**, **g** Transwell assay to assess the migration (**f**) and invasion (**g**) of MCF10A cells with FRDM3 knockdown and T47D, MDA-MB-231 cells with FRMD3 overexpression (left) and quantification of the indicated cell numbers (right). Scale bar, 100 μm. After 24 h, Migrated (**f**) and invaded (**g**) cells were counted following staining with crystal violet. **h** Representative images (top) of 3D spheroid invasion assay in MCF10 cells with FRMD3 knockdown and T47D, MDA-MB-231 cells with FRMD3 overexpression and quantification of 3D spheroid invasion area (bottom). Scale bar, 50 μm. The arrowhead pointed to the filopodium. All experiments were performed independently three times. *N* = 3 biologically independent replicates. The student’s *t*-test was used to estimate the significance of the difference between the two groups. Data were presented as means ± s.d. *P < 0.05; **P < 0.01; ***P < 0.001.

To explore the potential roles of FRMD3 in breast cancer, we ectopically overexpressed FRMD3 in T47D and MDA-MB-231 cell lines (with low FRMD3) by stable transfection of the FRMD3-expressing vector (pcflag-FRMD3) or knockdown FRMD3 in the MCF10A cell line (with high FRMD3) using lentivirus expressing shRNA and GFP. The stable overexpression of FRMD3 was confirmed in the indicated cell lines (Fig. 1l), and the MCF10A cell line with a stable FRMD3 knockdown or control shRNA was verified via GFP fluorescence detection and Western blot (Fig. 1m). Afterwards, we first tested the effect of FRMD3 on breast cell proliferation. The MTT assay and BrdU incorporation assay showed that FRMD3 overexpression significantly inhibited the proliferation of T47D and MDA-MB-231 cells while the FRMD3 knockdown promoted MCF10A cell proliferation (Fig. 2a, b). In the meantime, the colony formation assay also showed that FRMD3 overexpression dramatically inhibited the colony formation of BRCA cells whereas the MCF10A cells with the FRMD3 knockdown formed more as well as larger clones than the control cells (Fig. 2c). In addition, the soft agar assay for colony formation demonstrated that the FRMD3 overexpression greatly restrained the sphere formation of the T47D and MDA-MB-231 cells, whereas FRMD3 knockdown did not induce sphere formation in MCF10A cells (Fig. 2d). This result indicated that FRMD3 loss was not sufficient to trigger anchorage-independent growth in soft agar of normal mammary epithelial cells. Of note, the analysis of the tumor cell cycle and apoptosis using flow cytometry did not show apparent alterations (Fig. S1c–d). These data suggested a suppressive role for FRMD3 in BRCA cell proliferation and tumorigenesis.

### FRMD3 suppressed BRCA cell migration and invasion in vitro

**Fig. 3:**
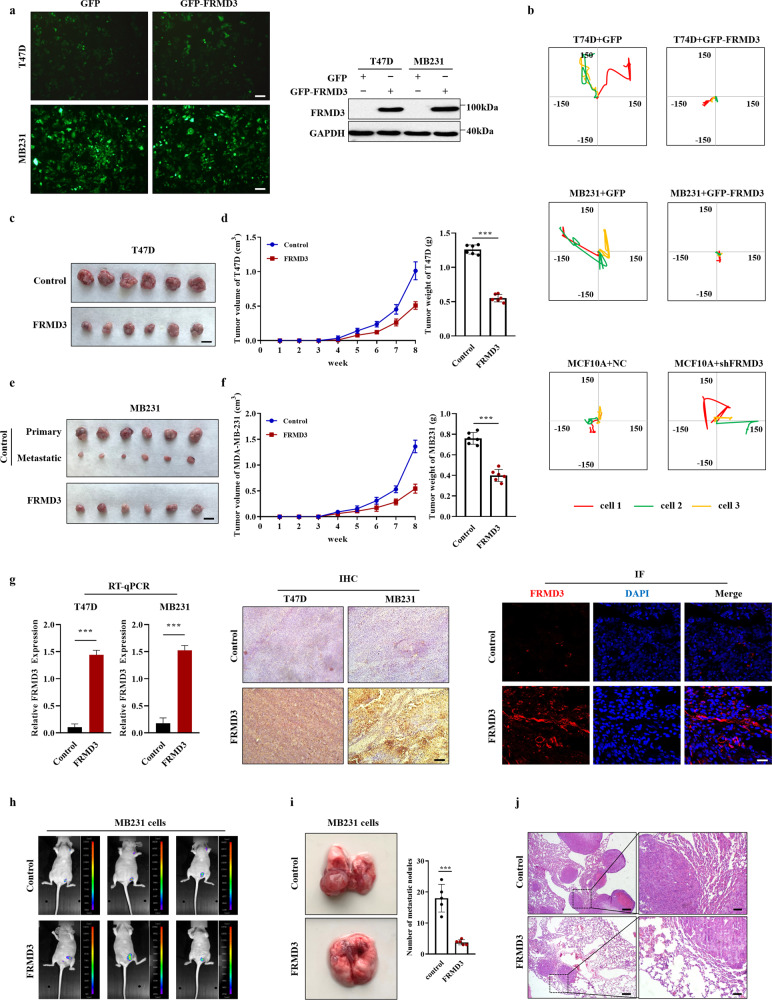
FRMD3 decreased the mobility of BRCA cells and their growth and metastasis in vivo. **a** GFP fluorescence microscopy (left) and Western blot (right) analysis of FRMD3 levels of T47D and MDA-MB-231 cell lines stably overexpressing GFP-FRMD3 established using lentivirus. Scale bar, 100 μm. **b** Cell tracking assay on three cells of MCF10 cells with FRMD3 knockdown or BRCA cells with GFP–FRMD3 overexpression, respectively. Images were captured every hour, and the trajectories of representative cells are plotted. The origins of migration are superimposed at (0, 0). **c**–**f** T47D (**c**, **d**) and MDA-MB-231 (**e**, **f**) cell lines stably overexpressing FRMD3-GFP, along with control stable cells, were injected subcutaneously into the mammary fat pads of female nude mice (*n* = 6 per group). Gross pictures (**c**, **e**), volumes (left in **d**, **f**) and weight (right in **d**, **f**) of xenografts were shown. Scale bar, 1 cm. **g** RT-qPCR (left), IHC (middle) and IF (right) analysis of the mRNA or protein levels of FRMD3 in the xenografts. Scale bar, 100 μm. **h** Representative in vivo GFP fluorescence images of primary and metastatic tumors derived from MDA-MB231 cells with FRMD3-GFP overexpression. Scale bar, 100 μm. **i** Representative images (left) of the lungs from BALB/c nude mice 30 days after tail vein injection with stable MDA-MB-231 cells with or without FRMD3-GFP overexpression and the number of metastatic nodules on the surface of the lungs from each group (right). (*n* = 6 per group). **j** Representative images of H&E staining of mouse lung tissues in Fig. 3i. Scale bars: 200 μm (left panel) and 50 μm (right panel). *N* = 3 biologically independent replicates. The student’s *t*-test was used to estimate the significance of difference between two groups. Data were presented as means ± s.d. ***P < 0.001.

Next, we further explored the effects of FRMD3 on BRCA cell migration and invasion. Cell scratch wound-healing (Fig. 2e) and transwell assays (Fig. 2f) exhibited that the ectopic expression of FRMD3 significantly inhibited the migration ability of BRCA cells. In contrast, FRMD3 knockdown markedly enhanced the metastatic potential of MCF10A cells. Then, we determined whether FRMD3 affected tumor cell invasion. The Matrigel invasion assay (Fig. 2g) and 3D spheroid invasion assay (Fig. 2h) showed that the invasion of T47D and MDA-MB-231 cells was blocked by FRMD3 overexpression, and FRMD3 knockdown augmented the invasion of MCF10A cells. In addition, we established the T47D and MDA-MB-231 cell lines stably overexpressing FRMD3-GFP using lentivirus as being capable of tracing cell migration in vitro and tumor growth and metastasis in vivo. The stable cell lines were verified by GFP fluorescence detection and Western blot (Fig. 3a). Then, we conducted the cell timelapse tracking assay to observe single-cell directional migration patterns. At 24 h after seeding cells at low density, the cell migration routes were recorded per hour. We observed that the average migratory displacement and distance of BRCA cells with FRMD3 overexpression were shorter than those of the control (Fig. 3b). Consistently, MCF10A cells with FRMD3 knockdown showed markedly longer migratory displacement and distance (Fig. 3b). All these data clearly indicated that FRMD3 played an inhibitory role in BRCA cell migration and invasion, the two processes that are closely associated with cancer metastasis and progression.

### FRMD3 inhibited the growth and metastasis of breast cancer in vivo

To further explore the effects of FRMD3 on BRCA growth and progression in vivo, T47D and MDA-MB-231 cell lines stably overexpressing FRMD3-GFP, along with stable control cells, were injected subcutaneously into the mammary fat pad of female nude mice. After eight weeks of tumor growth in vivo, the mice were euthanized to evaluate tumor size and weight. As compared to the control group, the tumors from T47D or MDA-MB-231 cells with FRMD3 overexpression were much smaller and lighter (Fig. 3c–f). Simultaneously, we confirmed by RT-qPCR, IHC, and IF staining that the FRMD3 levels were much higher in the tumors formed from stable FRMD3-overexpressed BRCA cells than those in the stable control tumors (Fig. 3g). In vivo fluorescence imaging showed that all the primary tumors from the stable control MDA-MB-231 cells appeared to metastasize to other distal sites (presumably axillary lymph nodes [23]) (6/6) (Fig. 3h), and the metastatic tumors removed were shown in Fig. 3e. However, no visible metastasis was found in mice injected with MDA-MB-231 cells overexpressing FRMD3 (Fig. 3h), as well as in those mice injected with either the control or FRMD3-overexpression T47D cells (data not shown). Finally, we conducted metastasis assay by tail vein injection to exclude the influence of the burden of primary tumor on its metastasis. Equal amount of control or FRMD3-GFP overexpressed MDA-MB-231 cells (2 × 10^6^) were tail vein injected into female nude mice (designated as control mice and FRMD3-mice respectively). Then, a significant reduction in the size and number of metastatic BRCA nodules was observed on the surface of the lungs of FRMD3-mice, compared with control mice (Fig. 3i). Specifically, the lungs of control mice were almost completely occupied by tumor nodules, resulting in losing the normal lung shape and being fragile of the residue lung tissue (Fig. 3i). Subsequent H&E staining further confirmed that there were much less metastatic lesions and more tissue with normal structure in the lungs of FRMD3-mice in contrast to control mice (Fig. 3j). In conclusion, animal experiments further confirmed the strong suppressive role of FRMD3 in BRCA growth and metastasis.

### FRMD3 downregulated the levels of vimentin and focal-adhesion-related proteins

**Fig. 4:**
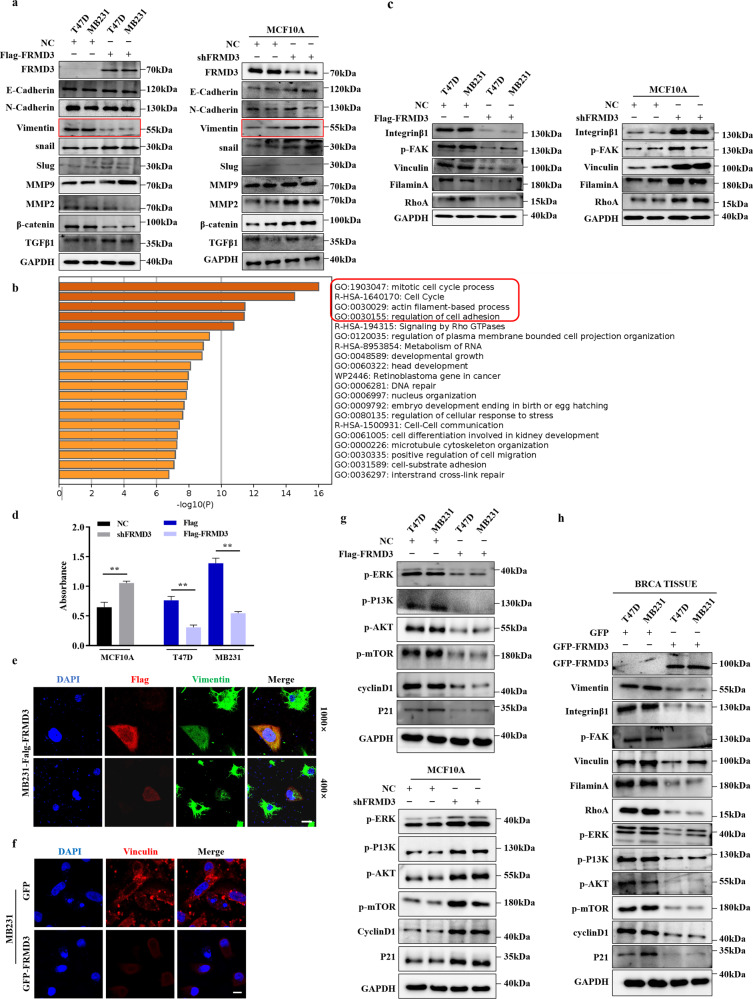
FRMD3 downregulated the expression of vimentin and focal adhesion related proteins. **a** Western blot analysis of EMT-related proteins in the indicated cells. **b** GO enrichment analysis of biological processes of FRMD3 positively associated genes in breast cancer. **c** Western blot analysis of the focal adhesion complex proteins in the indicated cells. **d** Cell adhesion analysis of MCF10 cells with FRMD3 knockdown and MDA-MB-231 cells with FRMD3 overexpression. The number of cells seeded in fibronectin-coated wells was determined by MTT assay. **e** Representative images of immunofluorescence staining of exogenous Flag-FRMD3 (red) and endogenous vimentin (green) in MDA-MB-231 cells transiently transfected with pc-flag-FRMD3 and photographed with a laser scanning confocal microscope under ×1000 (upper panel) and ×400 (lower panel) magnification. The nucleus was stained with DAPI (blue). Scale bars: 10 μm (upper panel) and 25 μm (lower panel). **f** Representative images of immunofluorescence staining of vinculin(red) in MDA-MB-231 cells with stable FRMD3 overexpression or control cells. The nucleus was stained with DAPI (blue). Scale bar, 25 μm. **g** Western blot analysis of proliferation-related proteins and signaling in indicated cells with overexpression or silencing of FRMD3. **h** Western blot analysis of the indicated proteins in xenografts shown in Fig. 3c and e. *N* = 3 biologically independent replicates. The student’s *t*-test was used to estimate the significance of difference between two groups. Data were presented as means ± s.d. **P < 0.01.

Next, we explored the molecular mechanism underlying FRMD3’s inhibitory effects on BRCA growth and progression. Epithelial-mesenchymal transition (EMT) plays a critical role in the invasion and metastasis cascades of cancer [24]; therefore, we first assessed the effects of FRMD3 on the EMT of BRCA cells. Unexpectedly, no significant changes in the expression of the epithelial marker (E-cadherin), the mesenchymal marker (N-cadherin), or the other EMT markers (e.g., slug, snail, MMP9, and MMP2) were observed in FRMD3-overexpression or knockdown cells, indicating there was no involvement of FRMD3 in the EMT process of BRCA cells (Fig. 4a and S2a). However, we noticed that the level of vimentin, one of the key EMT regulators and markers, was obviously downregulated upon FRMD3 overexpression in BRCA cells and consistently upregulated in FRMD3-knockdown MCF10A cells, as compared to control cells (Fig. 4a and S2a). To obtain clues for further studies, we selected the functional genes that are positively correlated with FRMD3 (Pearson’s correlation coefficient ≥ 0.5) from breast cancer in the UALCAN database for GO enrichment and biological process cluster analysis in Metascape. The results showed that FRMD3-related genes were significantly enriched in cell cycle, cell adhesion, and actin filament-based processes (Fig. 4b). Vimentin has been well documented to facilitate cancer invasion and metastasis via modulating EMT and focal adhesion [25]. Therefore, we examined the protein levels of the focal adhesion complex proteins [26]. As shown in Fig. 4c and S2b, β1-integrin, p-FAK, RhoA, vinculin and filamin A, the adaptor protein in focal adhesion, were all downregulated in the FRMD3-overexpressed BRCA cells, and FRMD3 knockdown in MCF10A cells obtained the opposite effects. Moreover, we also tested the adhesion abilities of cells and observed that the number of cells adhered to fibronectin (FN) significantly decreased in BRCA cells with FRMD3 overexpression but increased in FRMD3 knockdown MCF10A cells, as compared to control cells (Fig. 4d). Then the pcflag-FRMD3 plasmids were transiently transfected into MDA-MB-231 cells, and immunofluorescence staining showed that, as compared to FRMD3 negatively transfected cells (red fluorescence negative cells), FRMD3 positively transfected cells (red fluorescence stained) exhibited much weaker green staining of vimentin (Fig. 4e and S2c) and showed reductions in the number of extensions and the aggregation spike structures, leading to smoother perimembrane (Fig. 4e and S2d). Furthermore, we performed vinculin immunostaining in MDA-MB-231 cells with stable FRMD3 overexpression, which can show the focal adhesion plaques in the cells. We observed that FRMD3-overexpressed cells showed a much weaker staining of vinculin, which was in line with the Western blot results (Fig. 4f and S2e) and much less focal adhesions than the control cells (Fig. 4f and S2f). In general, FRMD3 overexpression decreased the levels of vimentin and related cytoskeleton and adhesion proteins, which could cause less cytoplasmic extensions, protrusion of the membrane and attenuated focal adhesions.

As for the possible mechanisms of FRMD3 inhibiting BRCA cell proliferation, we examined the activation of typical signaling pathways closely related to cancer growth and progression as well as the expression levels of representative cell cycle regulators in FRMD3 overexpression and knockdown cells. The results showed that FRMD3 overexpression strongly decreased while FRMD3 knockdown obviously elevated the levels of p-PI3K, p-AKT, p-mTOR, and p-ERK in BRCA cells and MCF10A cells, respectively, suggesting that FRMD3 negatively regulated the PI3K-AKT-mTOR and ERK pathways (Fig. 4g and S2g). At the same time, FRMD3 overexpression downregulated, while FRMD3 knockdown upregulated, the expression levels of cyclin D1 in corresponding cells, which agreed with the suppressive effects of FRMD3 on BRCA cell proliferation (Fig. 4g and S2g). In contrast, P21, generally considered a cell cycle inhibitor, was also downregulated by FRMD3 overexpression and upregulated by FRMD3 knockout. However, P21 has been reported to play dual roles as a tumor suppressor and an oncogene [27, 28]. In addition, the above effects of FRMD3 on signaling, cell cycle regulation, and focal adhesion complex proteins were further verified in T47D and MDA-MB-231 cell xenografts in mice, as shown in Fig. 4h and S2h. Overall, these data indicate that FRMD3 may suppress proliferation, metastasis, and aggressiveness in breast cancer cells through modulating cell adhesion, cell cycles, and a series of pathways.

### The ubiquitin-like domain of FRMD3 bound to vimentin, leading to its ubiquitination and degradation

**Fig. 5:**
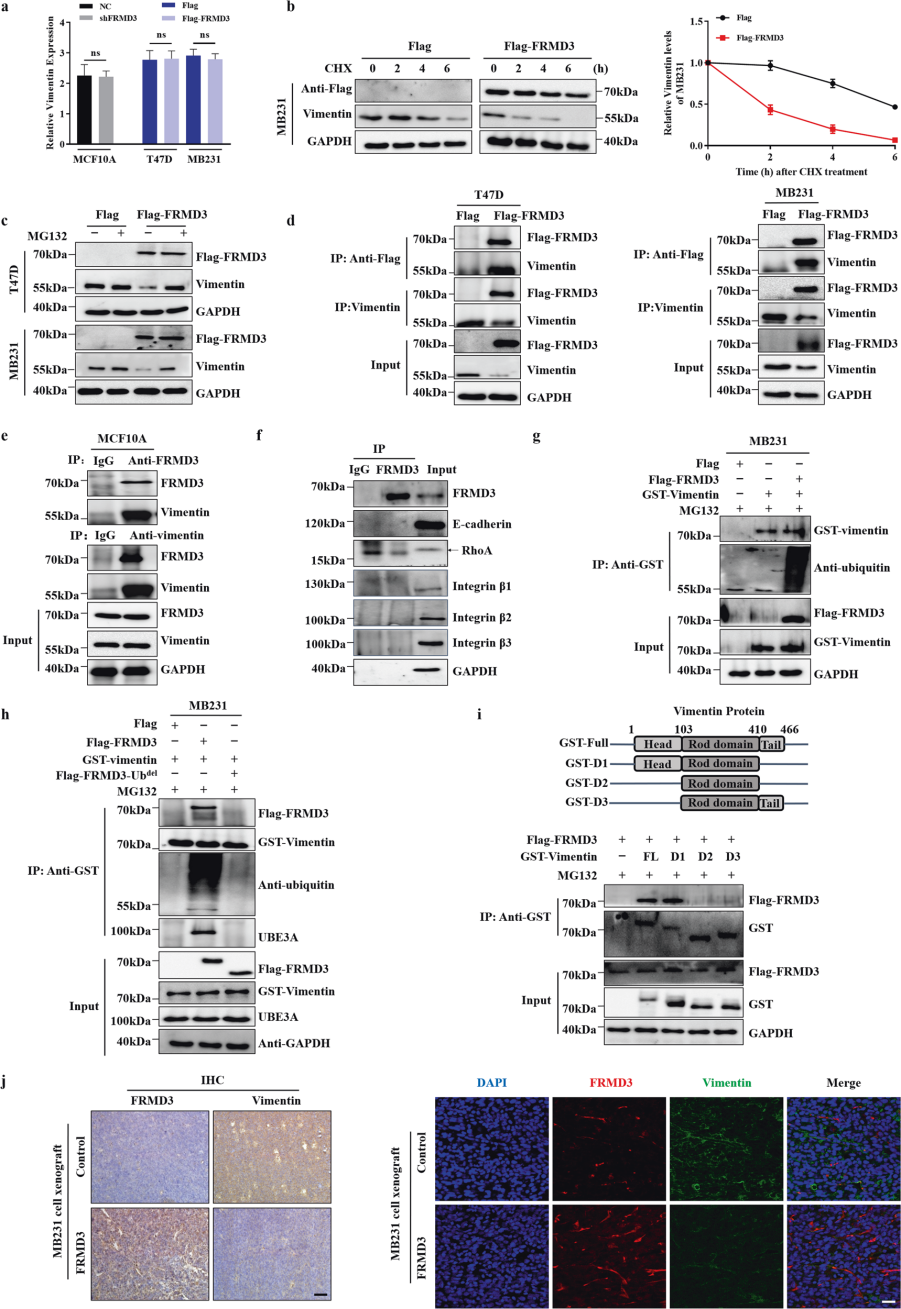
The ubiquitin-like domain of FRMD3 was required for the ubiquitination and degradation of vimentin through interaction with its head domain. **a** RT-qPCR analysis of the mRNA expression levels of vimentin in the cells with FRMD3 overexpression or knockdown. **b** Western blot analysis of the stability of the vimentin protein in control and FRMD3-overexpressing MDA-MB-231 cells in the presence of cycloheximide (CHX, 50 μg ml^−1^) for the indicated times (left). The data were quantified using ImageJ software (right), and GAPDH was used for normalization. **c** Western blot analysis of vimentin protein levels in control and FRMD3-overexpressed cells under the treatment with the proteasome inhibitor MG132 (10 μM for 8 h), or not. **d** Co-IP analysis of the interaction between FRMD3 and vimentin in T47D cells and MDA-MB-231 cells with FRMD3 overexpression. **e** Co-IP analysis of interaction between the endogenous FRMD3 and vimentin in MCF10A cells. Isotype-matched IgG was used as a negative control. **f** Co-IP analysis of the interaction of endogenous FRMD3 with the indicated proteins in MCF10A cells. **g** Immunoprecipitated ectopically expressed GST-vimentin from MG132-treated MDA-MB-231 cells with FRMD3 overexpression were subjected to Western blot with anti-ubiquitin antibodies. **h** Ectopically expressed GST-vimentin in MG132-treated MDA-MB-231 cells with FRMD3 or FRMD3-Ub^del^ overexpression were immunoprecipitated with anti-GST, and then the immunoprecipitated was subjected to Western blot with the indicated antibodies. **i** Co-IP analysis of the interaction of FRMD3 with different regions of vimentin in MG132-treated MDA-MB-231 cells co-transfected with pcFlag-FRMD3 and full-length vimentin (FL) or individual truncated vimentin mutants (schematic diagram shown on the top). **j** IHC (left) or immunofluorescence staining (right) analysis of the levels of FRMD3 and vimentin in MDA-MB-231 cell xenograft in nude mice shown in Fig. 3e. Scale bar, 100 μm. *N* = 3 biologically independent replicates. The student’s *t*-test was used to estimate the significance of difference between two groups. Data were presented as means ± s.d ns not significant.

Except for its well-known critical roles in cancer metastasis, vimentin is also involved in coordinating cell proliferation [29–31] and providing the platforms for cells to modulate signaling pathways, such as regulating ERK signaling [32]. Furthermore, it was reported that vimentin could associate with integrin to facilitate ERK activation by FAK [33]. Therefore, we focused on vimentin in this study. First, we explored how FRMD3 downregulated the vimentin levels in BRCA cells. We detected the mRNA levels of vimentin by RT-qPCR and found no significant change in vimentin mRNA levels under no matter FRMD3 overexpression or knockdown (Fig. 5a), which excluded the possibility of the transcriptional regulation of vimentin by FRMD3. Therefore, we speculated as to whether FRMD3 would promote the degradation of vimentin protein post-translationally. To this end, we measured the half-life of vimentin protein in BRCA cells treated with cycloheximide (CHX), a protein synthesis inhibitor. As compared to that in control cells, vimentin protein had a shorter half-life in FRMD3-ovexpressing T47D and MDA-MB-231 cells (Fig. 5b and S3a). Meanwhile, the decreased vimentin levels in FRMD3-overexpressed BRCA cells could be reversed by treatment with the proteasome inhibitor MG132 (Fig. 5c), indicating the involvement of the proteasome system in FRMD3-mediated vimentin degradation. Next, we tested the interaction between FRMD3 and vimentin by coimmunoprecipitation (Co-IP) in the three cell lines. As expected, Co-IP analysis revealed an interaction between FRMD3 and vimentin in BRCA cells (Fig. 5d) and MCF10A cells (Fig. 5e), respectively. Moreover, the immunofluorescence shown in Fig. 4e also indicated the co-localization of FRMD3 and vimentin on the cytoskeleton in MDA-MB-231 cells. It is noteworthy that we did not observe the interaction of FRMD3 with other relevant molecules detected in MCF10A cells (Fig. 5f). Considering that FRMD3 was predicted to have a ubiquitin-like domain, we next investigated whether FRMD3 mediated the ubiquitination of vimentin. Indeed, FRMD3 overexpression induced the polyubiquitination of vimentin in BRCA cells (Fig. 5g and S3b). Meanwhile, we hypothesized that the ubiquitin-like domain of FRMD3 could be responsible for the interaction with vimentin to promote its polyubiquitination. To this end, we generated a pcflag-FRMD3-Ub^del^ construct for the ectopic expression of FRMD3 with the deletion of the ubiquitin-like domain (FRMD3-Ub^del^). A Co-IP assay demonstrated that FRMD3-Ub^del^ could not bind to vimentin, and correspondingly, it could not mediate the ubiquitination of vimentin in MDA-MB-231 and T47D cells (Fig. 5h and S3c). Furthermore, the Co-IP assay also showed that the endogenous ubiquitin protein ligase E3A (UBE3A) could interact with vimentin when vimentin had been co-expressed with wild-type FRMD3 in MDA-MB-231 cells (Fig. 5h and S3c). However, when an empty vector or FRMD3-Ub^del^ was co-expressed with vimentin, vimentin could no longer interact with UBE3A (Fig. 5h and S3c). This result strongly suggests that FRMD3 may recruit vimentin and UBE3A for vimentin ubiquitination and degradation through its ubiquitin-like domain. In addition, phosphorylation and acetylation covalent modifications of vimentin were not obviously affected when either full-length FRMD or FRMD3-Ub^del^ was overexpressed in BRCA cells (Fig. S3d). To further identify the domain of vimentin contributing to the FRMD3 binding, three plasmids expressing the head domain, rod domain, and tail domain of vimentin were constructed and applied to the Co-IP assay in MDA-MB-231 cells. The results showed that FRMD3 was specifically bound to the head domain of vimentin but not to the rod and tail domains (Fig. 5i). Finally, we performed IHC staining to detect vimentin and dual immunofluorescence staining to detect both vimentin and FRMD3 on the xenograft tissue of MDA-MB-231 cells, with or without FRMD3 overexpression. We could observe the significant decrease in the vimentin level in an FRMD3-overexpressed xenograft, as compared to the control (Fig. 5j), further confirming the inverse relationship of the FRMD3 and vimentin levels. Overall, these data suggest that the ubiquitin-like domain of FRMD3 binding to the head domain of vimentin could induce polyubiquitination of vimentin, thereby enabling vimentin degradation through recruiting the ubiquitin ligase UBE3A.

### FRMD3 elicited its anti-tumor effects on BRCA via downregulation of vimentin

**Fig. 6:**
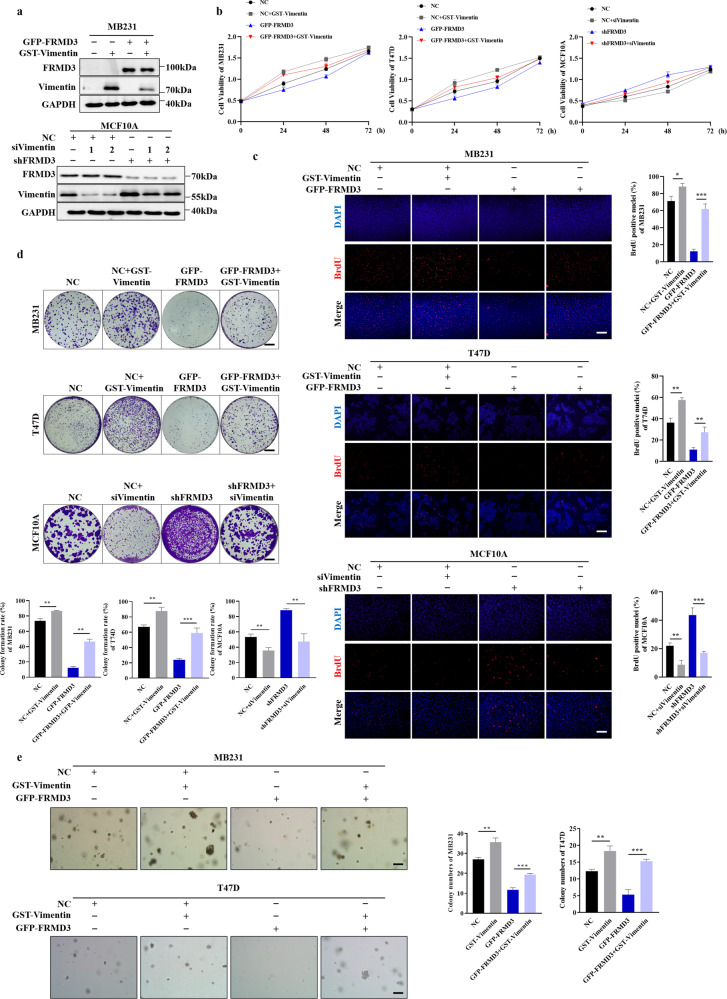
FRMD3 inhibited BRCA cell proliferation and soft agar colony formation by downregulation of vimentin. **a** Western blot analysis of the expression of FRMD3 and vimentin in stable FRMD3-overexpressed BRCA cells transfected with pcGST-vimentin plasmids or in stable FRMD3 knockdown MCF10A cells transfected with siRNA targeting vimentin (siVimentin). **b**–**d** MTT assay (**b**), BrdU incorporation assay (**c**), and plate colony formation assay (**d**) were performed to assess cell proliferation of the indicated cells. **e** Soft agar colony formation assay in T47D cells and MDA-MB-231 cells with FRMD3 overexpression, with or without vimentin overexpression. *N* = 3 biologically independent replicates. The student’s *t*-test was used to estimate the significance of difference between two groups. Data were presented as means ± s.d. *P < 0.05; **P < 0.01; ***P < 0.001.

**Fig. 7:**
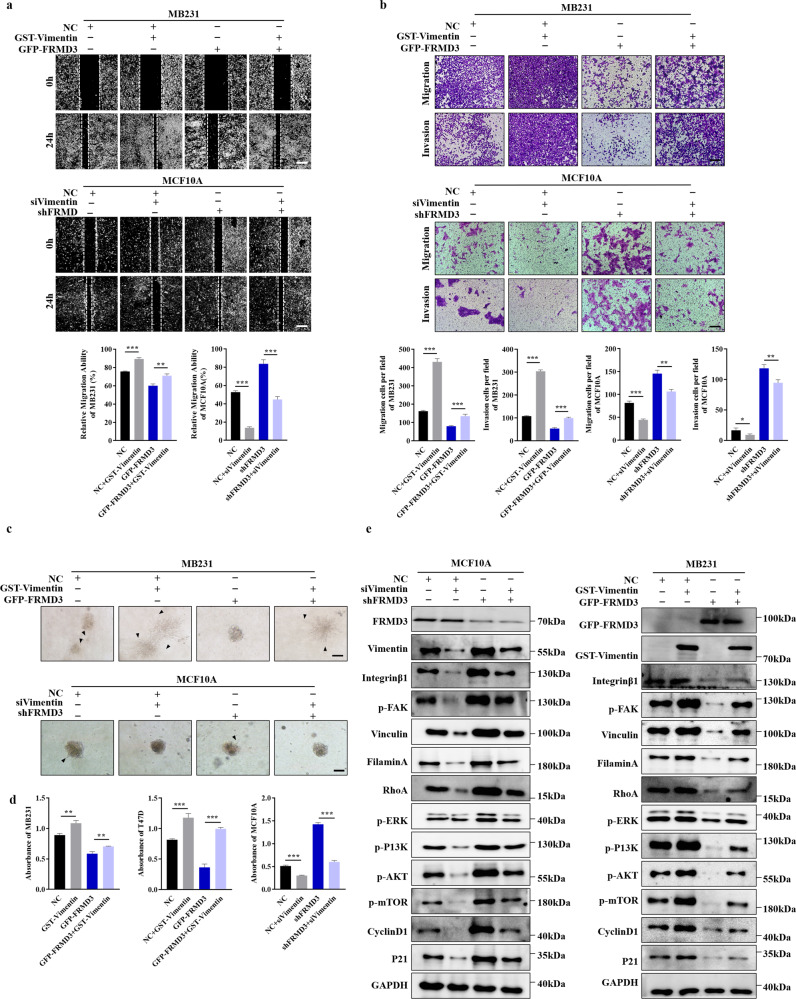
FRMD3 inhibited BRCA cell migration, invasion, and adhesion by downregulation of vimentin. **a**–**c** Scratch wound-healing assay (**a**), Transwell assay (**b**), and 3D spheroid invasion assay (**c**) were performed to detect cell migration and invasion in stable FRMD3-overexpressed MDA-MB-231BRCA cells transfected with pcGST-vimentin plasmids or in stable FRMD3 knockdown MCF10A cells transfected with siRNA targeting vimentin (siVimentin). **d** Cell adhesion analysis of indicated cells. MTT assay was used to quantify the cells adhered to fibronectin-coated wells. **e** Western blot analysis of the indicated proteins in corresponding cells with vimentin overexpression or knockdown. *N* = 3 biologically independent replicates. The student’s *t*-test was used to estimate the significance of difference between two groups. Data were presented as means ± s.d. **P < 0.01; ***P < 0.001.

To further explore whether FRMD3 inhibited BRCA growth and metastasis via interacting with, and the downregulation of, vimentin, we ectopically overexpressed vimentin in BRCA cells with stable FRMD3 overexpression and knocked down vimentin by siRNA in MCF10A cells with stable FRMD3 knockdown (Fig. 6a and S4a). The results showed that the overexpression of vimentin could completely or partially rescue the decreased proliferation, migration, and invasion caused by the FRMD3 overexpression in T47D and MDA-MB-231 cells (Figs. 6b–e, 7a–c and S4b–e). Consistently, the knockdown of vimentin blocked the enhancement of cell proliferation, migration, and invasion mediated by FRMD3 knockdown in MCF10A cell (Figs. 6b–e, 7a–c and S4b–e). In addition, we further demonstrated that the overexpression of vimentin reversed the FRMD3-mediated repression on the molecular events related to focal adhesion, signaling, and cell cycle in BRCA cells, as shown in Fig. 4 (Fig. 7d–e and S4f–g). In contrast, silencing vimentin restored the repression of these molecular events in MCF10A cells with stable FRMD3 knockdown (Fig. 7d–e and S4g).

### The anti-tumor effects of FRMD3 on BRCA cells required its ubiquitin-like domain

**Fig. 8:**
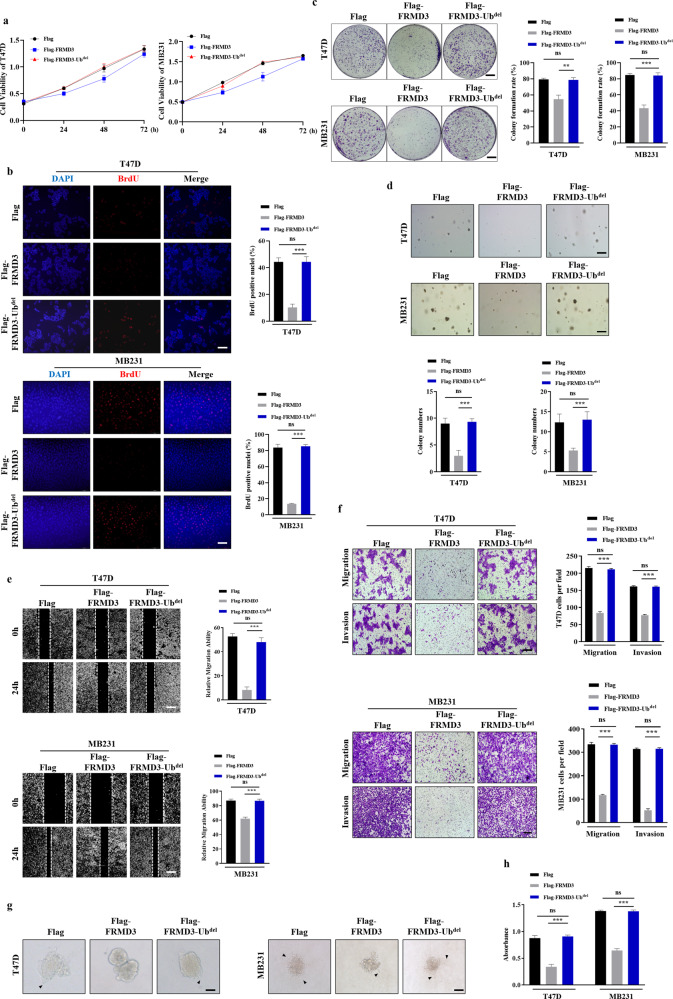
The ubiquitin-like domain of FRMD3 was indispensable for its anti-tumor effects on BRCA. **a**–**d** MTT assay (**a**), BrdU assay (**b**), plate colony formation assay (**c**), and soft agar colony formation assay (**d**) were performed to detect cells proliferation of T47D cells and MDA-MB-231 cells with overexpression of FRMD3, FRMD3-Ub^del^, or vector control. **e**, **f** The wound-healing assay (**e**), transwell migration and transwell Matrigel invasion assays (**f**) were conducted to assess migration and invasion of T47D cells and MDA-MB-231 cells overexpressing FRMD3, FRMD3-Ub^del^, or vector control. **g** Representative images of the 3D spheroid invasion assay in T47D cells and MDA-MB-231 cells overexpressing FRMD3, FRMD3-Ub^del^, or vector control. **h** Cells adhesion analysis of T47D and MDA-MB-231 cells overexpressing FRMD3, FRMD3-Ub^del^, or vector control. MTT assay was used to quantify the cells adhered to fibronectin-coated wells. *N* = 3 biologically independent replicates. The student’s *t*-test was used to estimate the significance of difference between two groups. Data were presented as means ± s.d. ns not significant; **P < 0.01; ***P < 0.001.

Considering that the ubiquitin-like domain of FRMD3 accounts for vimentin interaction, we then investigated the effects of FRMD3 with ubiquitin-like domain deletion on BRCA cells by transfecting pcflag-FRMD3-Ub^del^ into T47D and MDA-MB-231 cells, with vector or pcflag-FRMD3 transfection as controls. As compared to wild-type FRMD3, we found that ubiquitin-like domain-deleted FRMD3 could not inhibit BRCA cell proliferation (Fig. 8a–d), migration (Fig. 8e, f), invasion (Fig. 8f, g, S4h), and adhesion (Fig. 8h), which showed no significant difference to the vector control. These data suggest that the ubiquitin-like domain, or the vimentin binding domain, is indispensable for FRMD3 to elicit its anti-tumor effects on BRCA, which further verified that the downregulation of vimentin was at least one of the key mechanisms underlying FRMD3’s tumor suppressive effects on BRCA.

## Discussion

Metastatic dissemination of breast cancer cells remains a major obstacle to effective therapy. Metastasis consists of a series of sequential and interrelated steps: tumor cells first detach from the tumor mass, migrate across the adjacent tissue, invade the blood or lymphatic vessels, survive in the circulatory system, invade into a secondary site, and start to ectopically proliferate [34]. This process relies on the capability of cell invasion, mobility, and adhesion. In a systemic search for regulators of these steps, the loss of function of metastasis suppressor genes has been a significant rate-limiting step in breast cancer progression [35]. Here, we proposed that FRMD3 could inhibit the adhesion and motility of breast cancer cells by inducing the ubiquitination and degradation of vimentin, an essential regulator of cancer metastasis. Therefore, FRMD3 would act as a tumor suppressor in breast cancer, and its downregulation would be associated with poor clinical outcomes in breast cancer patients, indicating its potential as a prognostic marker and therapeutic target for breast cancer metastasis prevention and therapy.

In this study, we explored the downregulation of FRMD3 mRNA and its clinical relevance in breast cancer using the TCGA database and verified the lower levels of FRMD3 protein in both breast cancer cell lines and clinical tissue biopsy, as compared to normal controls. Then, overexpression and knockdown experiments demonstrated that FRMD3 suppressed BRCA cell proliferation, migration, and invasion in vitro (Fig. 2) and inhibited the in vivo growth and metastasis of BRAC cells in nude mice (Fig. 3). These data indicate that FRMD3 functions as a tumor suppressor in BRCA, which is in line with previous studies in lung cancer [7]. Interestingly, some members of the protein 4.1 superfamily have also been shown to act as tumor suppressors. For example, the loss of merlin expression was associated with the development of neurofibromatosis 2-related tumors and merlin functions as a growth negative regulator [36]; likewise, the expression of DAL-1/protein 4.1B was absent in a variety of human tumors, including non-small cell lung cancer and breast cancer, and DAL-1 was able to suppress cell proliferation and EMT and impair cell motility [37]. Merlin and DAL-1 may be associated with unique proteins that are specific to their function as a negative growth regulator in specific tissue [38]. Here, we showed that FRMD3 interacted with vimentin to suppress BRCA cell proliferation and metastasis via mediating vimentin degradation. Furthermore, we demonstrated that the ubiquitin-like domain, located at the N-terminus of the FERM domain, was not only responsible for vimentin binding, but also the sole functional domain for FRMD3’s inhibitory effects on BRCA since mutant FRMD3 with the deletion of the ubiquitin-like domain almost completely lost its tumor suppressor function in BRCA (Fig. 8). For most members of the protein 4.1 superfamily, there have been one or two additional conserved domains other than FEMD domain, that are essential for their functions. Additional biologic activities and functional domains of FRMD3 are expected to be revealed in future studies.

Vimentin is associated with poor prognoses and invasion of breast cancer, bladder cancer, esophageal cancer, lung cancer, and other cancers, and it is a potential target for cancer treatment [39–42]. Previous studies have shown that vimentin knockdown impaired cell attachment, migration, and invasion in breast and colon cancer cell lines [43]. In this study, we also demonstrated that vimentin was essential for the proliferation, migration, and invasion of BRCA cells with downregulated FRMD3, as the ectopic expression of vimentin could reverse the inhibition of BRCA cell proliferation, migration, and invasion by FRMD3. Consistently, knockdown vimentin abolished the enhanced proliferation, migration, and invasion of MCF10A normal mammary epithelial cell due to the FRMD3 silence (Figs. 6, 7). Several lines of evidence have suggested that polyubiquitination may be one of the critical post-translational modifications that mediated vimentin degradation. For example, misfolded vimentin in muscle cells had been recognized and guided to the proteasome for degradation by a ubiquilin2 (UBQLN2) and myotubularin-1 (MTM1) complex that ensures cytoskeletal integrity [44]. In ovarian cancer cells, E3 ubiquitin ligase TRIM56 induced the multi-ubiquitin-mediated proteasome degradation of vimentin, leading to reduced cell migration and invasion [45]. Moreover, Trim16-dependent vimentin polyubiquitination and degradation was reported in lung adenocarcinoma that was diminished by AKT-induced lncRNA VAL [20]. Specifically, in triple-negative breast cancer (TNBC), RING finger protein 208 (RNF208), an estrogen-inducible E3 ligase, targeted soluble vimentin for polyubiquitin-mediated proteasomal degradation, thereby suppressing metastasis [39]. Our data demonstrated that FRMD3 bound with the head domain of vimentin and recruited the ubiquitin ligase UBE3A to induce the polyubiquitination and degradation of vimentin, in which no phosphorylation or acetylation was found to be involved.

Though FRMD3 leads to vimentin downregulation, the suppressive role of FRMD3 in BRCA growth and metastasis is irrelevant to EMT as we demonstrated that FRMD3 did not affect the EMT process. Instead, FRMD3 could lead to the general downregulation of the focal adhesion complex proteins including β1-integrin, p-FAK, RhoA, vinculin and filamin A (Fig. 4c), following the induction of vimentin degradation while the overexpression of vimentin could restore the levels of these proteins in FRMD3-overexpressed BRCA cells (Fig. 7d). The downregulation of the focal adhesion complex proteins by FRMD3 led to cytoskeleton rearrangement and defects in the cell membrane protrusion of BRCA cells (Fig. 4f, g). Vimentin interacts with the β1-intergrin cytoplasmic tail domain to promote integrin recycling, motility, and activation, leading to FAK phosphorylation [33]. Activated FAK activates downstream pathways including PI3K-AKT-mTOR and ERK signaling to regulate cell migration, proliferation, and survival [46, 47]. Indeed, PI3K-AKT-mTOR and ERK signaling were both repressed by FRMD3 in BRCA cells (Fig. 4h). In addition, though one 4.1 superfamily member 4.1 G was shown to directly bind β1-integrin with its FERM domain and increase its affinity to the ligand [48], we failed to demonstrate the interaction between FRMD3 and β1-integrin. Notably, cytoskeletal protein aberrations are the underlying cause of many pathological phenotypes. In fact, a number of diseases other than cancers have been associated with abnormalities of cytoskeletal and nuclear skeletal proteins, including cardiovascular disease, neurodegenerative disorders, and cirrhosis [49, 50]. Therefore, whether the regulation of FRMD3 on vimentin or focal adhesion could underlie the close association of FRMD3 gene variation and diabetic nephropathy could be worthy of further investigation.

Finally, FRMD3 is poorly expressed in BRCA cells but the underlying mechanisms are still unclear. As both the mRNA and protein levels of FRMD3 were downregulated in our study, we first speculated as to whether epigenetic mechanisms were involved. We preliminarily detected the DNA methylation of the FRMD3 promoter in BRCA cells and MCF10A cells respectively; however, no difference was observed (data not shown). Recently, FRMD3 was found to be the most downregulated protein by hsa-miR-3651, a novel predictor for in-breast recurrence [9]. Therefore, it may be likely that FRMD3 is downregulated in BRCA through miRNA-mediated silence, but other mechanisms such as gene mutations or lost and the regulation of long non-coding RNA (lncRNA) may also be involved, which should be investigated in future studies. Moreover, further comprehensive studies are needed to explore whether FRMD3 is downregulated in other malignant tumors, other than BRCA, and its functional roles.

In conclusion, our results suggest a mechanism by which FRMD3 interacts with vimentin and ubiquitin ligase UBE3A through its ubiquitin-like domain to promote proteasome degradation of vimentin, thereby exerting a unique function of FRMD3 in breast cancer progression. Meanwhile, FRMD3 inhibits integrin synthesis by degrading vimentin, resulting in cytoskeletal rearrangement and reduced focal adhesions and affecting the regulation of downstream proteins on tumor cell tumorigenesis, metastasis, and aggressiveness (Fig. 8i). These results may contribute to a better understanding of the role of FRMD3 and vimentin in breast cancer metastasis and provide a direction for new therapeutic approaches in the development of targeted drugs and inhibitors for breast cancer.

## Methods

### Cell culture and plasmid or siRNA transfection

MCF10A, T47D, MDA-MB-231, ZR7530, and HEK-293T cells were obtained from the Chinese Academy of Sciences. All cells were authenticated and tested for mycoplasma contamination. MCF10A cells were cultured in DMEM/F-12 supplemented with 10% fetal bovine serum (FBS) (TBD Science, Tianjin, China). T47D, MDA-MB-231 and ZR7530 cells were cultured in RPMI 1640 medium with 10% FBS. The HEK-293T cells were cultured in H-DMEM medium supplemented with 10% FBS. All mediums were supplemented with 100 units/mL penicillin and 100 mg/mL streptomycin. Cells were maintained at 37 °C in incubator containing 5% CO_2_. The cell lines in this study were not found in the database of commonly misidentified cell lines maintained by ICLAC and NCBI Biosample. All cells were authenticated and tested for mycoplasma contamination.

Recombinant pcDNA3.1-Flag vectors expressing full length or ubiquitin-like domain deleted FRMD3 and vimentin were constructed and synthesized by GenScript Biotechnology Co., Ltd. (Nanjing, Jiangsu, China). When the cell confluence reached 80%, plasmids were transfected into indicated cells using Lipofectamine 2000 Transfection Reagent (11668019, Thermo Fisher Scientific, Waltham, MA, USA) according to the manufacturer’s protocol. For stable transfection, the transfected cells were subsequently selected with G418 (200 µg/mL) for 2 weeks.

For siRNA Transfection, the siRNAs targeting vimentin (siRNA1 sense: GCAGAAGAAUGGUACAAAUTT; anti-sense: AUUUGUACCAUUCUUCUGCTT. siRNA2 sense: GACCUGCUCAAUGUUAAGATT; anti-sense: UCUUAACAUUGAGCAGGUCTT) were designed and synthesized by GenePharma (Suzhou, Jiangsu, China) and transfected into target cells by Lipofectamine 2000 Transfection Reagent according to the manufacturer’s protocol.

### Tumor xenograft studies and Bioluminescence imaging analysis

All animal studies were conducted with approval from the Institutional Animal Care and Use Committee of Northeast Normal University (NENU/IACUC, AP20191225) of China, and all experiments conform to the relevant regulatory standards. Female BALB/c nude mice (4–6 weeks old) were purchased from Charles River Animal Company of China and raised under specific-pathogen-free (SPF) conditions. Nude mice were randomly and blindly assigned to experimental groups (*n* = 6 in each group).

For xenograft experiments, equal numbers of the established stable cells were injected subcutaneously into the mammary fat pad of female nude mice. Mice were anaesthetized using isoflurane before being subjected to in vivo imaging system (NightOWL LB 983, Berthold, Germany) every week. 8 weeks after injection, the mice were euthanized, and tumor volumes and weight were measured with a caliper and calculated using the equation, volume = 1/2 × ab^2^ (a = length, b = width). Then some tumors together with mouse lungs and livers were fixed with 4% formaldehyde and embedded in paraffin. Other tumors were frozen in −80 °C for SDS-PAGE and Western blot analysis.

For tail vein assay of cancer metastasis, stable cells (2 × 10^6^) were injected into the tail vein of nude mice. After 30 days, mice were euthanized and sacrificed. The lungs were dissected and photographed. Then the tumor nodules on the surface of the lungs were separately counted by two researchers, followed by lung fixation in 4% formaldehyde and embedding in paraffin.

The embedded tissues were serially sectioned and subjected to hematoxylin and eosin (H&E) staining (G1121, Solarbio, Beijing, China) according to the manufacturer’s protocol and immunohistochemical (IHC) staining (see below), which were observed under an Olympus BX50 microscope (Olympus, Tokyo, Japan).

### Bioinformatics analysis

In the cancer genome atlas (TCGA) database, samples of breast cancer were selected for an analysis of FRMD3 expression and further used for bioinformatics analysis on The University of Alabama at Birmingham cancer data analysis Portal (UALCAN) network. Then, Gene Expression Profiling Interactive Analysis (GEPIA) database (http://gepia.cancer-pku.cn/) was applied to perform survival analysis based on 532 samples with high expression of FRMD3 and 531 samples with low expression of FRMD3. Kaplan–Meier curves were generated using the Kaplan–Meier plotter.

### Antibodies and reagents

Anti-FRMD3 (Cat # 53575) was purchased from Invitrogen. Anti-ubiquitin (Cat #sc8017), anti-cyclin D1 (Cat # sc-751), anti-cyclin B1 (Cat # sc-752), and anti-cyclin E (Cat # sc-198) were from Santa Cruz Biotechnology (Santa Cruz, CA, USA). Anti-vimentin (Cat #10366), anti-flag (Cat #20543), anti-GST (Cat #66001), anti-vinculin (Cat #26520), anti-FLNA (Cat #67133), anti-UBE3A (Cat #10344), anti-E-cadherin (Cat #20874), anti-N-cadherin (Cat #22018) anti-integrinβ1 (Cat #26918) was obtained from Protein-Tech Group. Anti-Phospho-FAK (Cat # 8556) were from Cell Signaling Technology (Danvers, MA, USA). The mouse monoclonal antibody against GAPDH was purchased from Kangcheng Bio-tech (Shanghai, China). The 4,6-diamidino-2-phenylindole (DAPI) and cell cycle analysis kits were obtained from Beyotime (Shanghai, China). The cell proliferation ELISA kit (Bucculite™ FdU Cu-Free Cell Proliferation, BrdU) was purchased from ATT Bioquest (USA). The 3-(4,5-dimethylthiazol-2-yl)-2,5-diphenyl-2Htetrazolium bromide (MTT) was purchased from Sigma-Aldrich (St. Louis, USA).

### RT-qPCR detection and Western blot analysis

Total RNA was isolated using TRIzol reagent (15596026, Invitrogen, Waltham, MA, USA) and reverse-transcribed into cDNAs with EasyScript First-Strand cDNA Synthesis SuperMix (AE301-02, TransGen Biotech, Beijing, China), according to the manufacturer’s protocols.

The cDNA templates were then subjected to RT-qPCR using the SYBR Green PCR Kit (04913914001, Roche, Mannheim, Germany), according to the manufacturer’s instructions. Specific primers were designed for RT-qPCR detection (Supplementary Table 1) and were all synthesized and purchased from GENEWIZ Biotechnology Co., Ltd. (Suzhou, Jiangsu, China). The assay was performed with a PikoReal 96 PCR System (Thermo Fisher Scientific, Waltham, MA, USA). β-Actin was used as an internal control. The relative expression levels of the target genes were determined using the 2 − ΔΔCt method using PikoReal 2.1 software (Thermo Fisher Scientific, Waltham, MA, USA). Each sample was analyzed in triplicate.

The proteins were extracted with RIPA buffer (P0013B, Beyotime, Shanghai, China) and a nuclear-cytoplasmic extraction kit (P0028, Beyotime, Shanghai, China), separated by sodium dodecyl sulfate-polyacrylamide gel electrophoresis (SDS–PAGE), and transferred to polyvinylidene fluoride (PVDF) membranes (88518, Thermo Fisher Scientific, Waltham, MA, USA). The membranes were blocked with 5% skim milk (P0216, Beyotime, Shanghai, China) and then incubated in the primary antibodies with suggested dilutions. After washing, the membranes were incubated with HRP-conjugated anti-IgG (Proteintech, Wuhan, Hubei, China) for 2 h at room temperature. The antigen–antibody complexes on the membranes were detected with high-sig ECL reagent (180-5001, Tanon, Shanghai, China). The images were acquired using the MicroChemi system (70-25-00, NDR Bio-Imaging Systems, Jerusalem, Israel) and quantified with ImageJ software (ImageJ 1.50i, National Institutes of Health, Bethesda, MD, USA).

### Transwell migration assay

For migration assays, 8 μm diameter pore transwell cell culture inserts (BD Biosciences) were placed in six-well plates. The underside of the insert and the bottom of the well were coated with 10 μg/ml of FN at 37 °C for 1 h. Cells suspended in serum-free media were seeded into the upper chamber of the insert (4 × 10^5^/well), and complete medium was added to the lower chamber. Cells were then incubated for another 6 h, during which cells migrated through the pores in the insert to the lower side of the membrane insert. At the end of cell migration, we cleansed the upper side of the chamber with a cotton swab, stained the filter for 1 h with crystal violet (C0121, Beyotime, Shanghai, China) in 2% ethanol, and then rinsed it in water. The filters were then imaged with an Olympus BX50 microscope. Five representative images (×10 magnification) were captured randomly for each insert and used to manually count the cells. The results were presented as mean number of cells per field ± S.D.

### Spheroid invasion assay in a three-dimensional (3D) culture

The 3D culture model was established as described previously [51]. Briefly, 1 × 10^4^ cells were suspended with Matrigel (356234, Corning, New York, NY, USA) and collagen type I (354236, Corning, New York, NY, USA) and kept at 37 °C until gelled. Then, the polymers were embedded in 24-well plates for 30 min at 37 °C to generate 3D culture systems, followed by submergence of the 3D cultures in the cell culture medium. After 48 h, images of invading cells were captured using an Olympus BX50 fluorescence microscope, and the invasion area was quantified using ImageJ software to calculate the invasion area of each spheroid.

### Wound healing assay

Cells were seeded with 5 × 10^5^ cells in six-well plate for 24 h in normal culture medium. Cells were arrested mitotically by incubation with 8 μg/mL mitomycin C (MCE) for 2 h under normal culture conditions. Mitomycin C was removed by three washes in PBS. A pseudo-wound was introduced in an equivalent confluent monolayer of cells by lightly scratching the cell layer with a 200 μl pipette tip. Cell debris was removed by two washes with culture medium. After 18 h, images were captured on an Olympus BX50 microscope. The wounded area was measured using ImageJ software for each representative time point.

### MTT assay and BrdU assay

Cell viability was determined by 3-(4, 5-dimethyl2-thiazolyl)-2, 5-diphenyl-2-H-tetrazolium bromide (MTT) assay. A total of 2000 cells per well were seeded in a 96‐well tissue culture plate. Then, the cells of each group were transfected. A total of 24, 36, 48, and 72 h after transfection, MTT assays were performed using a detection kit (Beijing Solarbio Science & Technology Co., Ltd.), according to the manufacturer’s protocol. For BrdU analysis, the medium was incubated with BrdU labeling solution for 3 h. After 48 h transfection, BrdU uptake was measured, according to the manufacturer’s protocol (Bucculite™ FdU Cu-Free Cell Proliferation, ATT Bioquest, USA). Images were captured using Olympus BX50 fluorescence microscope.

### Cell motility assay

A total of 5000 cells were seeded in 96-well plate and cultured in an incubator with humidified air (95%) and 5% CO_2_ at 37 °C for 6 h. Then the dynamics of cell movement was monitored using BD pathway (USA, 855) and scanned sequentially every 60 min for 20 h. At least three randomly chosen cells per group were monitored and presented.

### Co-immunoprecipitation (Co-IP)

Co-IP was performed using Protein A/G Magnetic Beads (HY-K0202, MedChemExpress, Monmouth Junction, NJ, USA), according to the manufacturer’s protocols, to examine protein-protein interactions. A total of 25 μL of magnetic beads pretreated with 0.5% Triton X-100 in PBS (PBST) solution were fully suspended with PBST containing the antibodies at a final concentration of 5 μg/mL and incubated at 4 °C for 2 h in a flip mixer. Proteins extracted from cells using IP lysis solution (87787, Thermo Fisher Scientific, Waltham, MA, USA) were fully suspended with the antibody–magnetic-bead complexes and incubated at 4 °C overnight in a flip mixer (88881002, Thermo Fisher Scientific, Waltham, MA, USA). After thorough washing, the antigen–antibody–magnetic-bead complexes were boiled in 25 μL of 1 × SDS–PAGE loading buffer, and the supernatant was subjected to Western blot analysis.

### Immunofluorescence (IF) detection

Cells were seeded on sterile coverslips in 6-well culture plates. After 24 h, the cells were fixed in 4% paraformaldehyde and washed for three times with PBS. Then, 0.1% Triton X-100 was used to permeate cells at room temperature for 5 min, and non-specific sites were then blocked with 5% bovine serum albumin for 30 min. Thereafter, primary antibodies were flooded over the cells, and incubated at 4 °C overnight. After extensive washing, the cells were incubated with FITC or HRP-conjugated secondary antibody for 1 h in the dark at room temperature. Nuclei were stained with DAPI for 5 min at room temperature. Finally, fluorescence was observed under a laser scanning confocal microscope and quantified using ImageJ software (LSM 880, ZEISS, Oberkochen, Germany).

### Immunohistochemical (IHC) staining

Tissue arrays containing multiple human breast cancer and pericarcinomatous tissues (HBreD055CD01and HBreD080CS01) were obtained from Shanghai Outdo Biotech Company (Shanghai, China). The clinical breast cancer tissue microarrays and mice tissue sections were analyzed by IHC staining, as previously described [52]. According to the protocols of the two-step detection kit (PV-9001, ZSGB-BIO, Beijing, China), the tissue microarray (TMA) slides or tissue sections were incubated in an appropriate endogenous peroxidase blocker for 10 min at room temperature, followed by incubation with anti-FRMD3 antibodies overnight at 4 °C and subsequent incubation with response enhancer and enhanced enzyme-labeled goat anti-rabbit IgG polymer for 20 min at room temperature. A DAB Chromogenic Kit (ZLI-9017, ZSGB-BIO, Beijing, China) was used to detect antibody binding, and the reaction was stopped by immersing the TMA slides or tissue sections in running water once a brown color appeared. Finally, the TMA slides or tissue sections were counterstained with hematoxylin (G1121, Solarbio, Beijing, China), dehydrated using a series of graded alcohol solutions, and mounted. Images were photographed with an Olympus BX50 microscope. Appropriate positive and negative controls were included for each run of the IHC assay.

### Lentiviral packaging and stable infection

Human FRMD3 cDNA or shRNA template targeting FRMD3 (shFRMD3 core sequence: GCUGGAGAAGGACUACUUUTT) was cloned into Plvx-AcGFP-N1 or pGreenPuro vectors respectively to construct recombinant expression lentivectors. The empty vector was used as the negative control. The expression lentivectors together with packaging genome plasmids pMD2G and pSPAX2, were co-transfected into 293T cells using Lipofectamine2000 Transfection Reagent. After 48 h, the viral supernatant was collected and filtered by 0.45 μm filter. The target cells were incubated with virus-containing supernatant for 12–16 h in the presence of 10 μg/mL polybrene (C0351, Beyotime, Shanghai, China). The infected cells were then selected with puromycin (2 μg/mL) (ST551, Beyotime, Shanghai, China) for 48 h.

### Statistical analysis

IBM SPSS Statistics was used for statistical analysis. The data were presented as mean ± S.D. All data were from three independent experiments. *P*-values were calculated from two-sided Student’s *t*-test or one-way analysis of variance (ANOVA); *P < 0.05, **P < 0.01, and ***P < 0.001 were displayed as statistically significant or not significant (ns).

## Supplementary information

Supplementary legends

figure S1

figure S2

figure S3

figure S4

Original western blots

checklist

## Data Availability

The datasets generated and/or analyzed during the current study are available from the corresponding author on reasonable request.
