# Supplementary material for: FRMD3 inhibits the growth and metastasis of breast cancer through the ubiquitination-mediated degradation of vimentin and subsequent impairment of focal adhesion
Source: Cell Death Dis. 2023 Jan 11;14(1):13. doi: 10.1038/s41419-023-05552-2 (PMC9834407; doi:10.1038/s41419-023-05552-2)
Supplement: Supplementary file 1 — Supplementary legends [file 41419_2023_5552_MOESM1_ESM.docx]

**Figure 1S. The expression of FRMD3 in BRCA cell lines and tissue and its effect on BRCA cell cycle, apoptosis and invasion**

a. The quantification of Western blot (Fig.1i) detecting the expression levels of FRMD3 in MCF10A normal breast epithelial cell line and different BRCA cell lines. b. Immunoreactivity score of FRMD3 protein levels in paracancerous tissues (n=14), luminal breast cancer tissues (n=33), HER^+^ breast cancer tissues (n=13) and triple negative breast cancer (TNBC) tissues (n=28). c-d. Flow cytometry analysis of cell cycle (c) and apoptosis distribution (d) of MCF10A cells with FRMD3 knockdown or T47D and MDA-MB-231 cells with FRMD3 overexpression.

*N*= 3 biologically independent replicates. The student’s t-test was used to estimate the significance of difference between two groups and more than two groups were analyzed by one-way ANOVA. Data were presented as means ± s.d. ns, not significant; **P* < 0.05; ***P* < 0.01; *** *P* < 0.001.

**Figure 2S. FRMD3 inhibited the expression of vimentin and focal adhesion related proteins.**

a. The quantification of Western blot analysis of EMT-related proteins in the indicated cells (Fig.4a). b. The quantification of Western blot analysis of the focal adhesion complex proteins in the indicated cells (Fig 4c). c. Relative fluorescence intensity of vimentin in transiently transfected MDA-MB-231 cells with or without pc-flag-FRMD3 (Fig.4e). d. The cell shape measurements which are expressed as the form factor, 4π(area)/(perimeter)^2^. e. Relative fluorescence intensity of vinculin in stably transfected MDA-MB-231 cells with or without FRMD3 overexpression (Fig.4f). f. The number of focal adhesions in stably transfected MDA-MB-231 cells with or without FRMD3 overexprssion based on vinculin immunostaining shown in Fig.4f. g. The quantification of Western blot analysis of proliferation-related proteins and signaling in indicated cells (Fig.4h). h. The quantification of Western blot analysis of the indicated proteins in xenografts shown in Fig.4i.

*N* = 3 biologically independent replicates. The student’s t-test was used to estimate the significance of difference between two groups. Data were presented as means ± s.d. **P* < 0.05; ** *P* < 0.01; *** *P* < 0.001.

**Figure 3S. The ubiquitin-like domain of FRMD3 was indispensable for the ubiquitination and interaction of vimentin.**

1. Western blot analysis of the stability of the vimentin protein in control and FRMD3-overexpressed T47D cells in the presence of CHX for the indicated time periods (left). The data were quantified using ImageJ software (right), and GAPDH was used for normalization. b. Immunoprecipitated ectopically expressed GST-vimentin in MG132-treated T47D cells with FRMD3 overexpression were subjected to Western blot with anti-ubiquitin antibodies. c. Ectopically expressed GST-vimentin in MG132-treated T47D cells with FRMD3 or FRMD3-Ubdel overexpression were immunoprecipitated with anti-GST, and then the immunoprecipitated was subjected to Western blot with the indicated antibodies. d. Immunoprecipitated ectopically expressed GST-vimentin from MG132-treated T47D or MDA-MB-231 cells with FRMD3 overexpression were subjected to Western blot with the indicated antibodies.

*N* = 3 biologically independent replicates. The student’s t-test was used to estimate the significance of difference between two groups. Data were presented as means ± s.d. ns, not significant; **P* < 0.05; ** *P* < 0.01; *** *P* < 0.001.

**Figure 4S FRMD3 restrained BRCA migration and invasion by down-regulating vimentin**

a. Western blot analysis of the expression of FRMD3 and vimentin in stable FRMD3-overexpressed T47D cells transfected with GST-vimentin plasmids. (b-d) The wound-healing assay (b), transwell migration, and Matrigel invasion assays (c) and 3D spheroid invasion assay (d) were conducted to detect cell migration and invasion in the indicated cells. e. The quantification of 3D spheroid invasion area in indicated cells (Fig.7c and S4d). f. Western blot analysis of the indicated proteins in T47D cells with vimentin overexpression or knockdown. g. The quantification of Western blot analysis of the indicated proteins in corresponding cells with vimentin overexpression or knockdown (Fig.7e and S4f). h. The quantification of 3D spheroid invasion area in T47D cells and MDA-MB-231 cells overexpressing FRMD3, FRMD3-Ub^del^, or vector control.

*N* = 3 biologically independent replicates. The student’s t-test was used to estimate the significance of difference between two groups. Data were presented as means ± s.d. **P* < 0.05; ** *P* < 0.01; *** *P* < 0.001.
