# Supplementary figures and images for: FRMD3 inhibits the growth and metastasis of breast cancer through the ubiquitination-mediated degradation of vimentin and subsequent impairment of focal adhesion
Source: Cell Death Dis. 2023 Jan 11;14(1):13. doi: 10.1038/s41419-023-05552-2 (PMC9834407; doi:10.1038/s41419-023-05552-2)

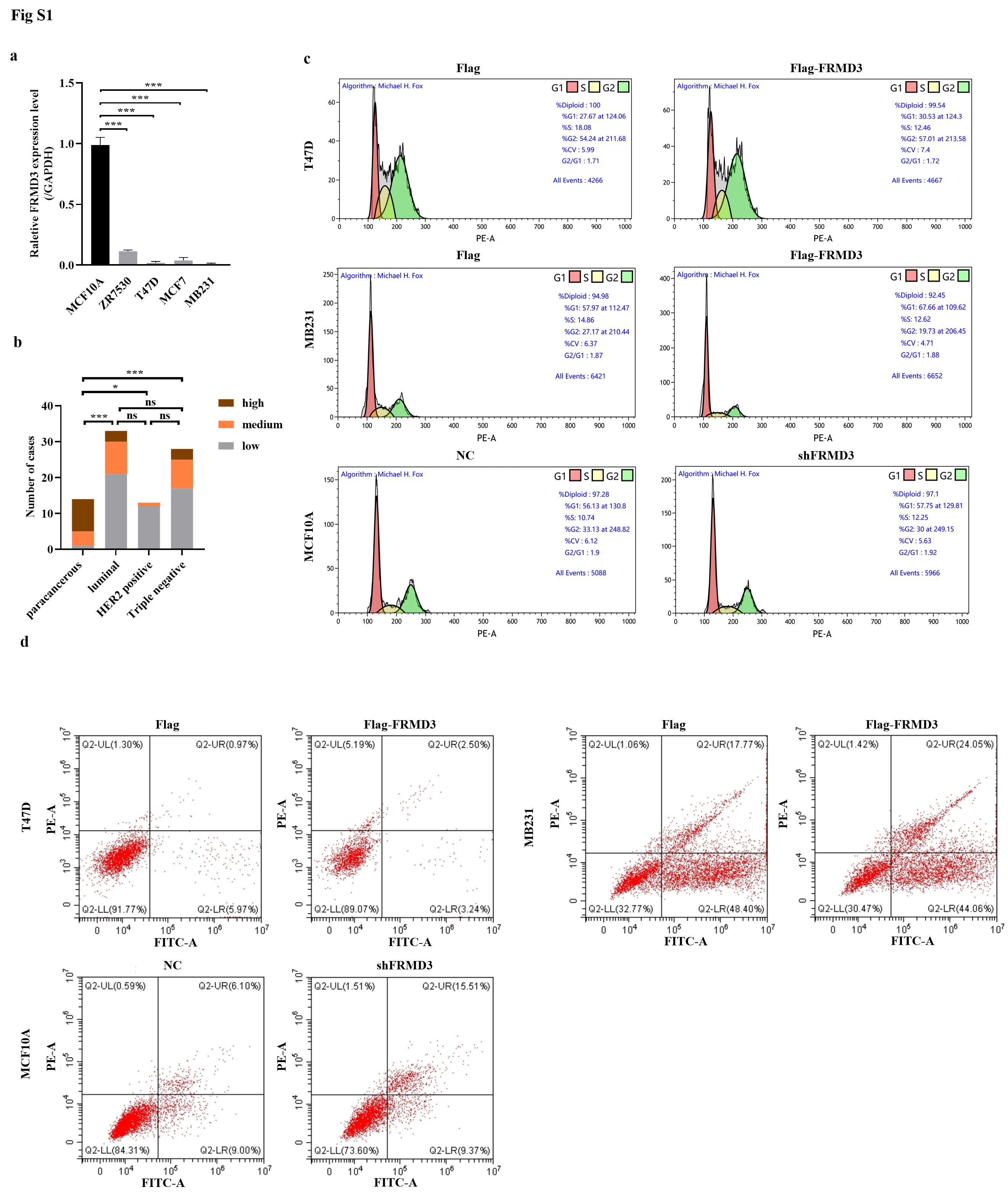

Supplement: Supplementary file 2 — figure S1 [file 41419_2023_5552_MOESM2_ESM.png]

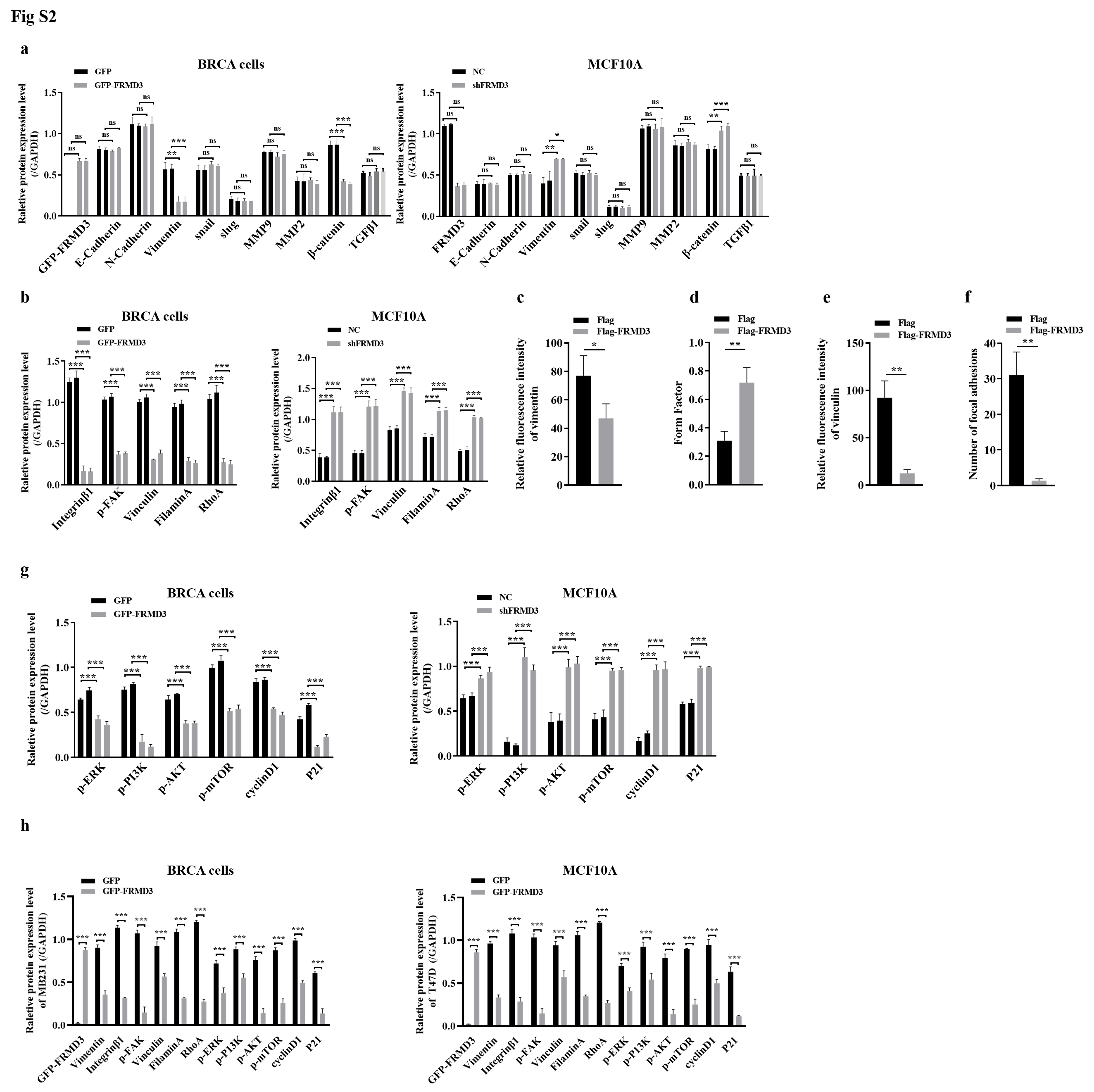

Supplement: Supplementary file 3 — figure S2 [file 41419_2023_5552_MOESM3_ESM.png]

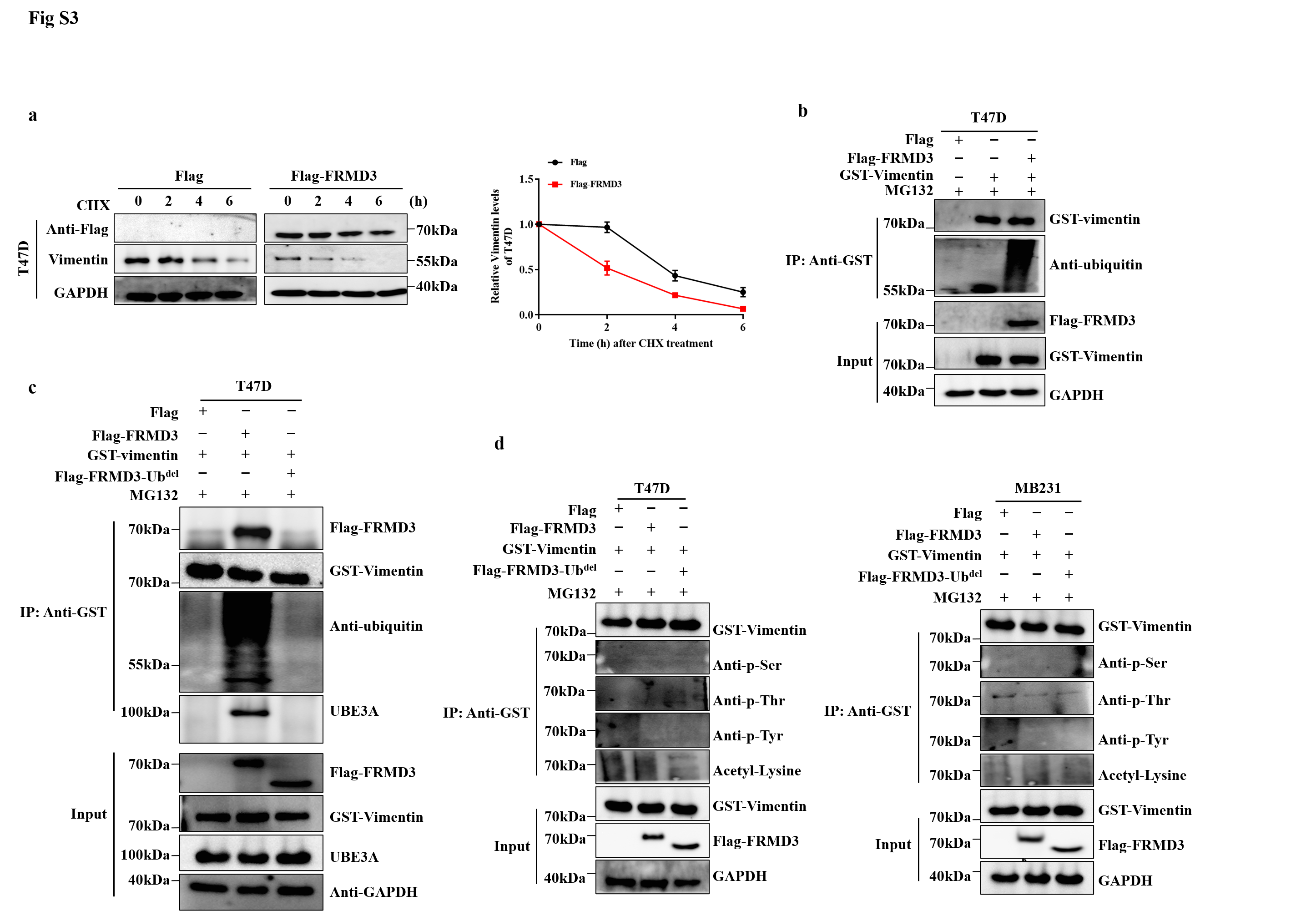

Supplement: Supplementary file 4 — figure S3 [file 41419_2023_5552_MOESM4_ESM.png]

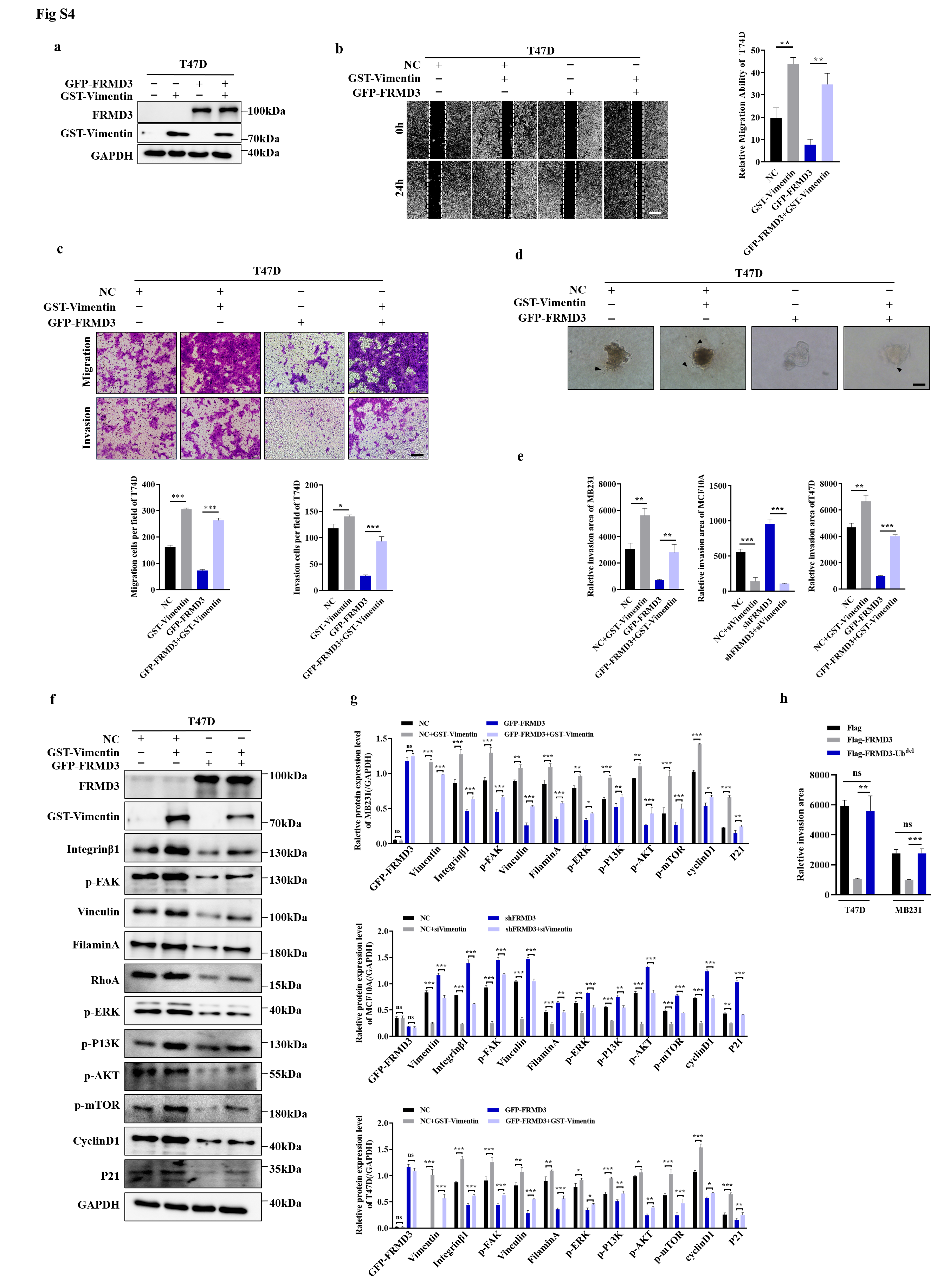

Supplement: Supplementary file 5 — figure S4 [file 41419_2023_5552_MOESM5_ESM.png]
