# Supplementary material for: FRMD3 inhibits the growth and metastasis of breast cancer through the ubiquitination-mediated degradation of vimentin and subsequent impairment of focal adhesion
Source: Cell Death Dis. 2023 Jan 11;14(1):13. doi: 10.1038/s41419-023-05552-2 (PMC9834407; doi:10.1038/s41419-023-05552-2)
Supplement: Supplementary file 6 — Original western blots [file 41419_2023_5552_MOESM6_ESM.pdf]

Original western blots

Fig 1i

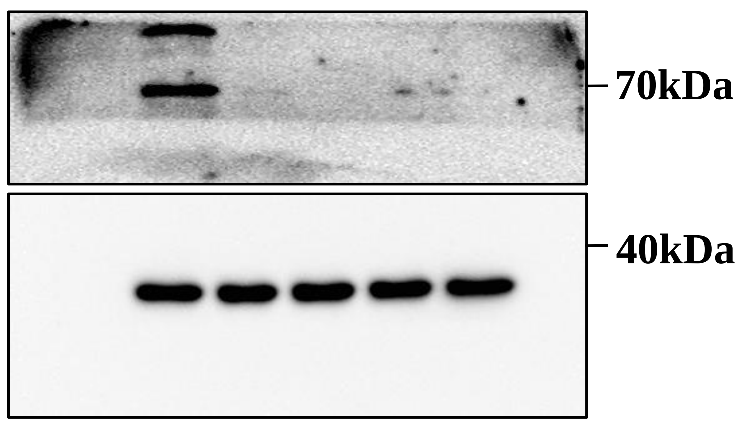

Fig 1l

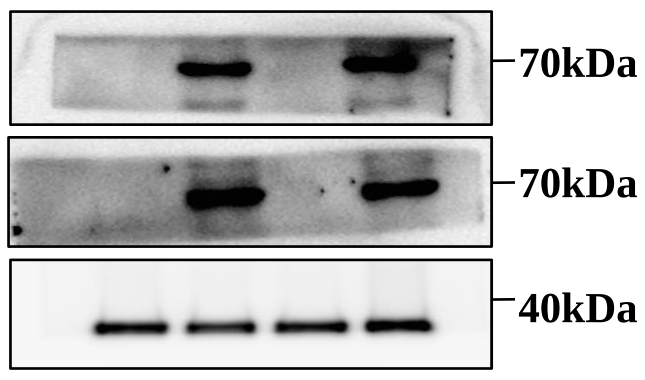

Fig 1m

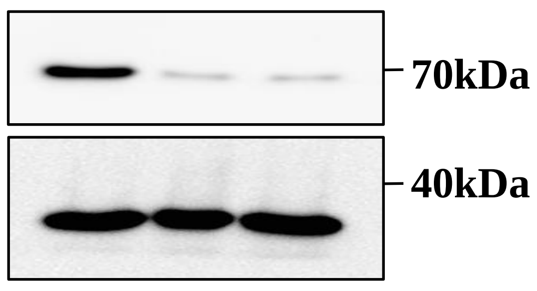

Fig.3a

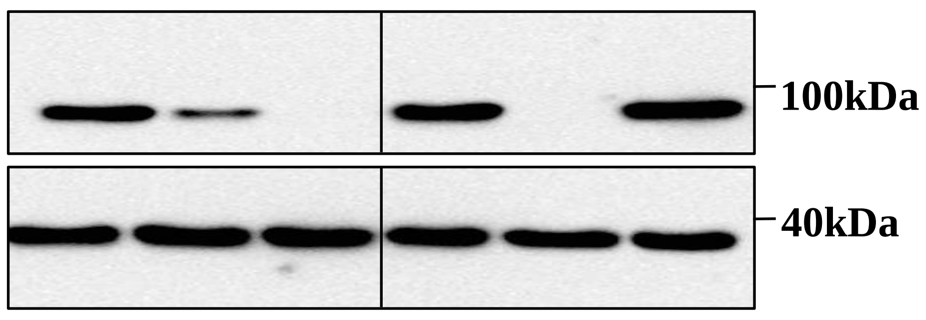

Fig 4a

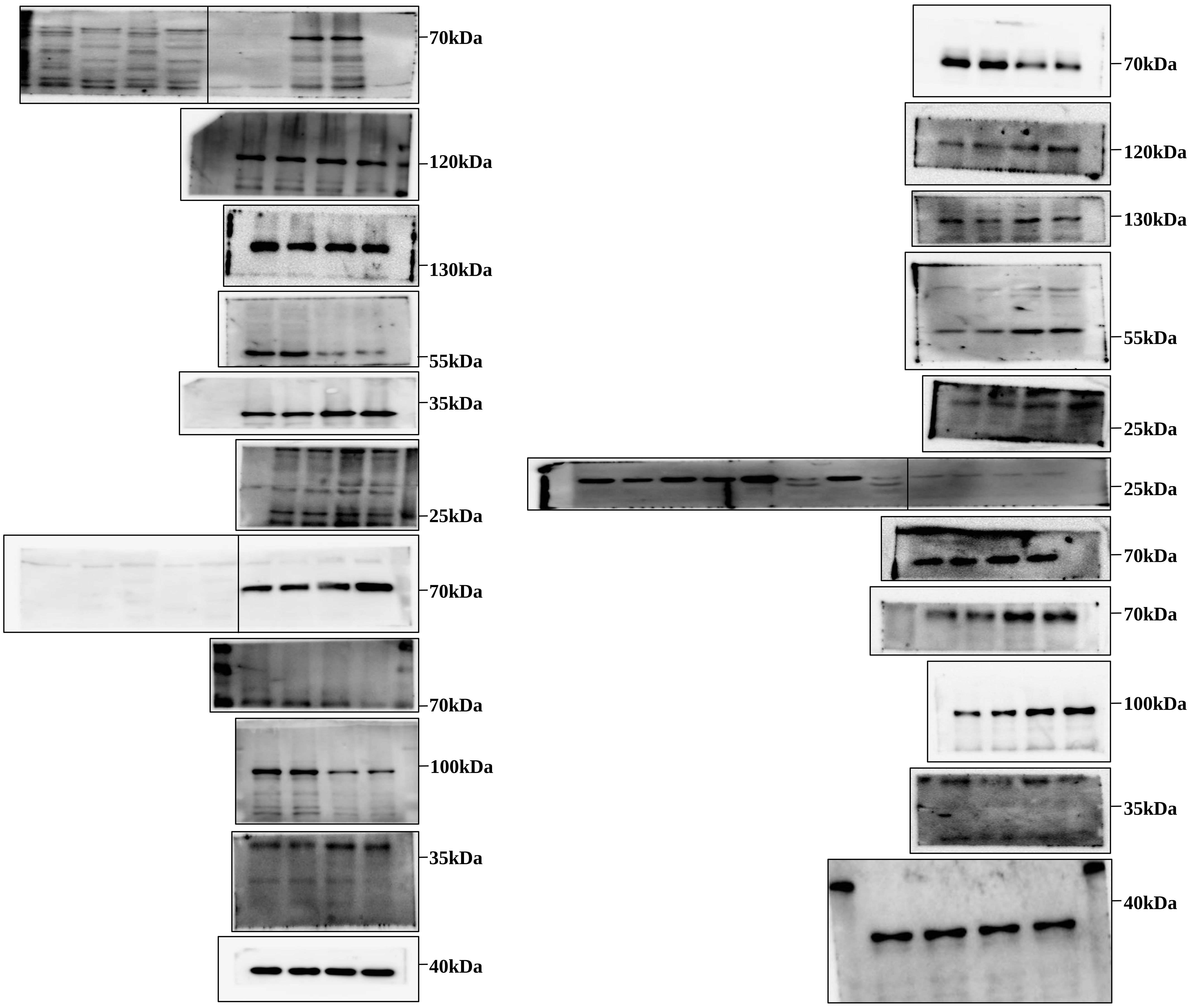

Fig 4c

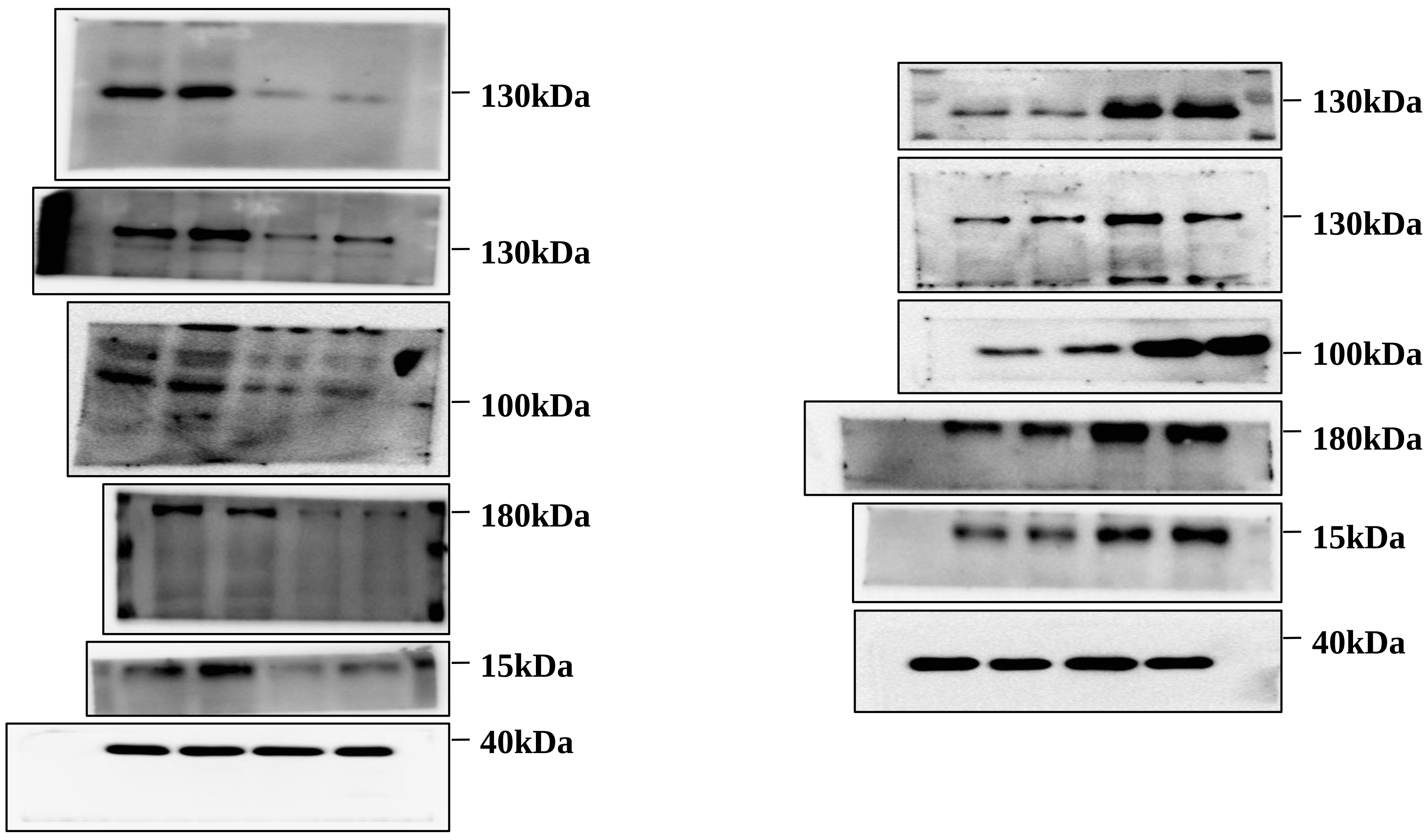

Fig 4g

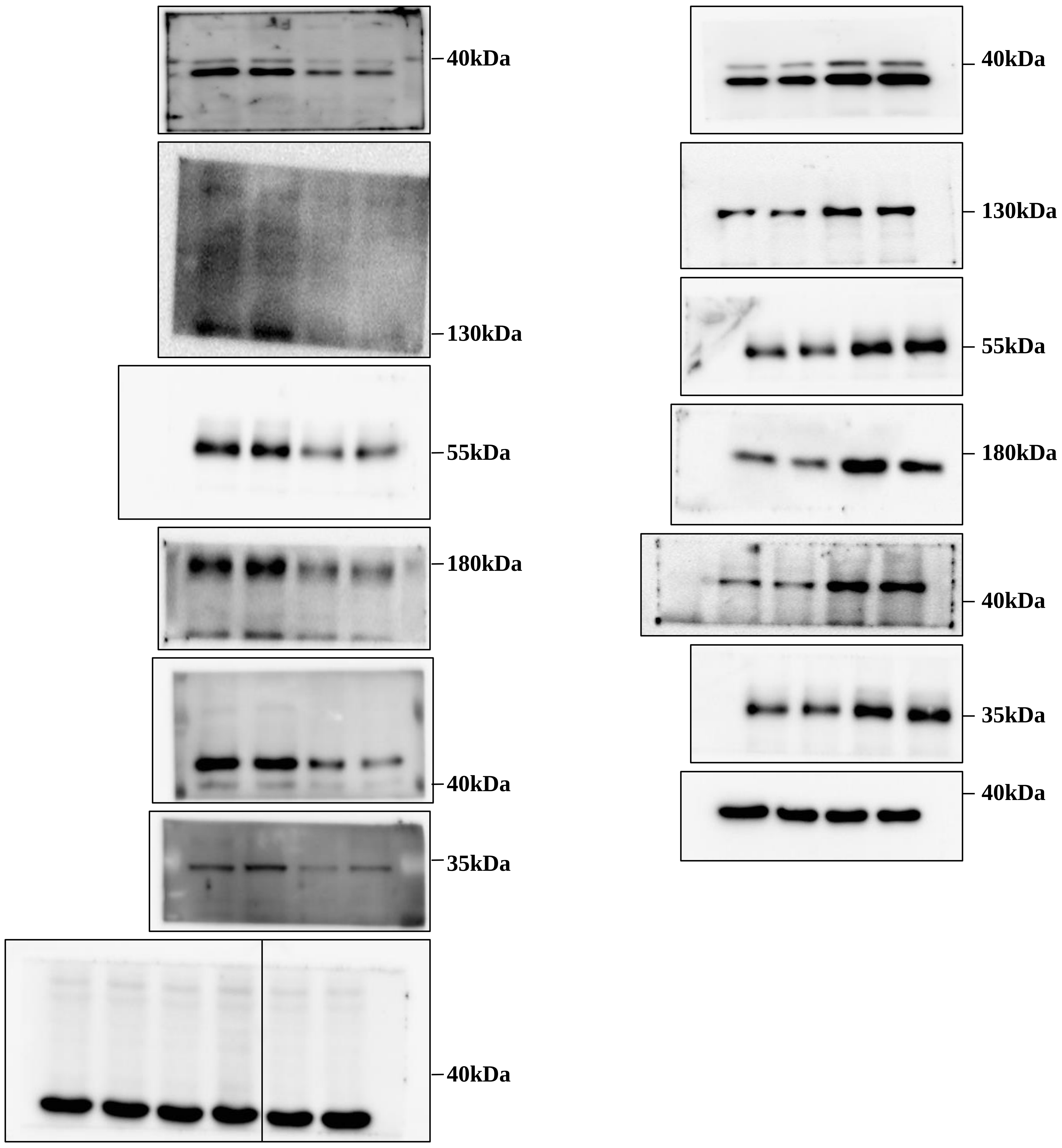

Fig 4h

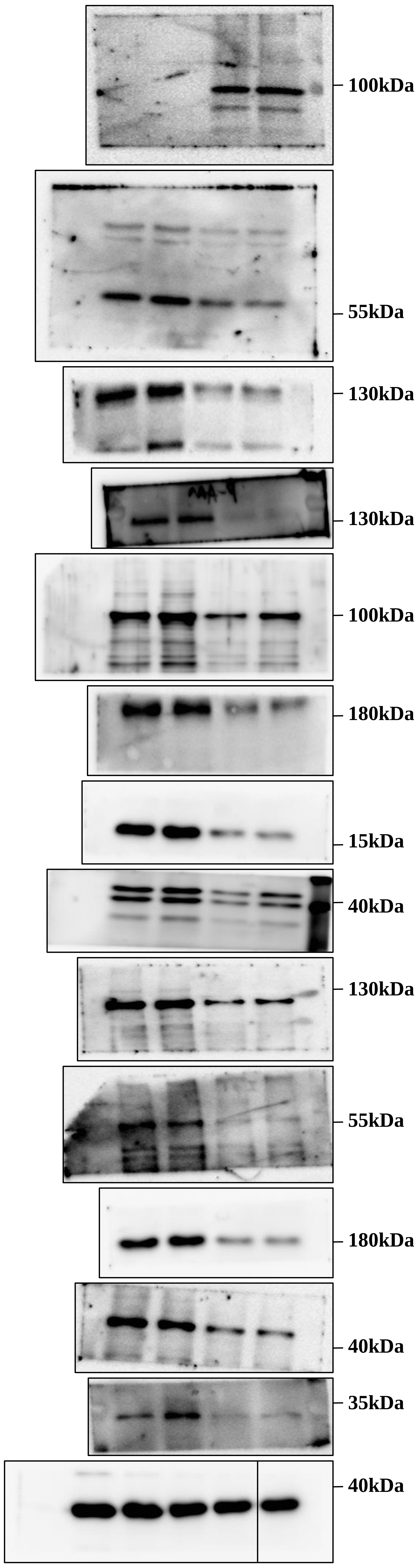

Fig 5b

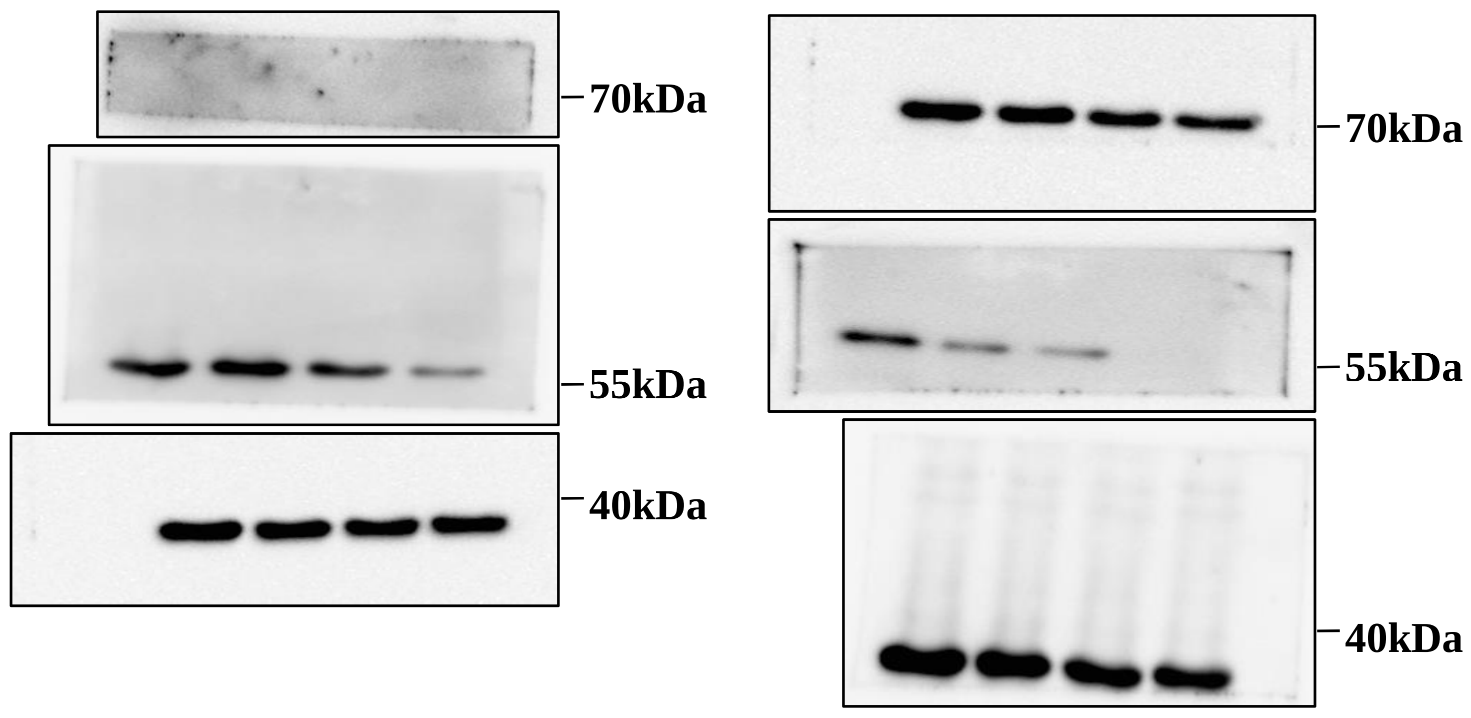

Fig 5c

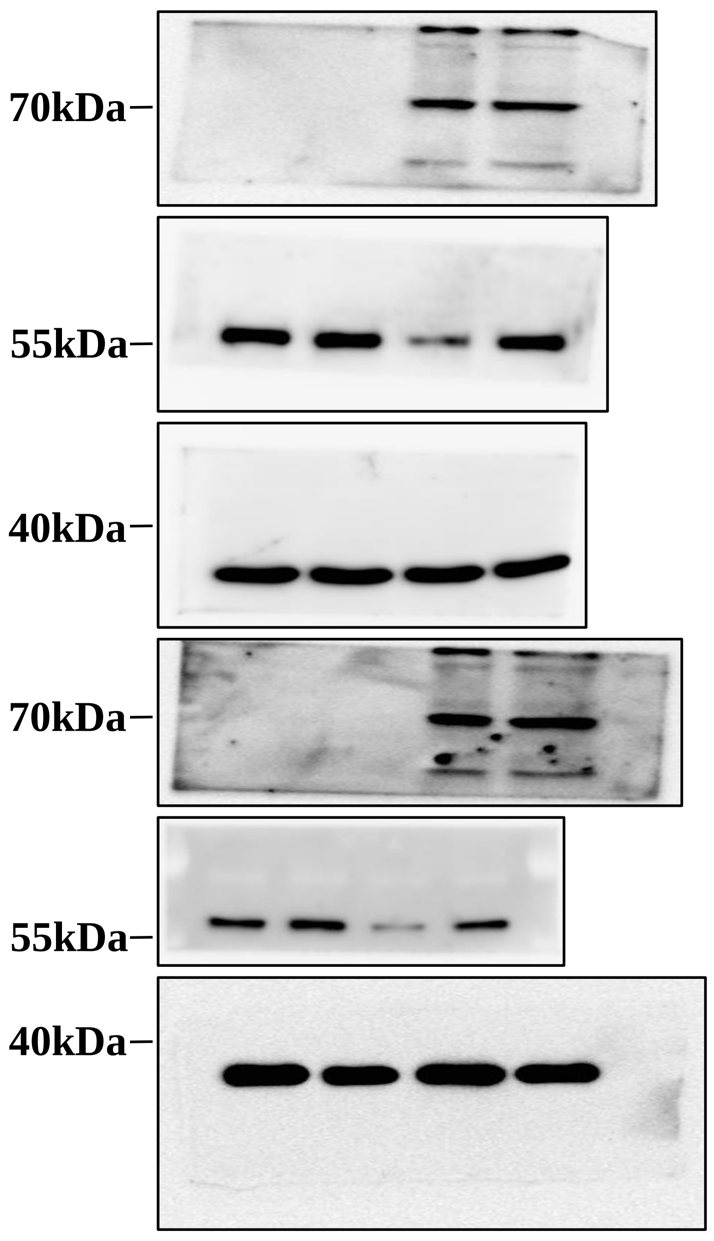
70kDa
55kDa
40kDa
70kDa
55kDa
40kDa

Fig 5d

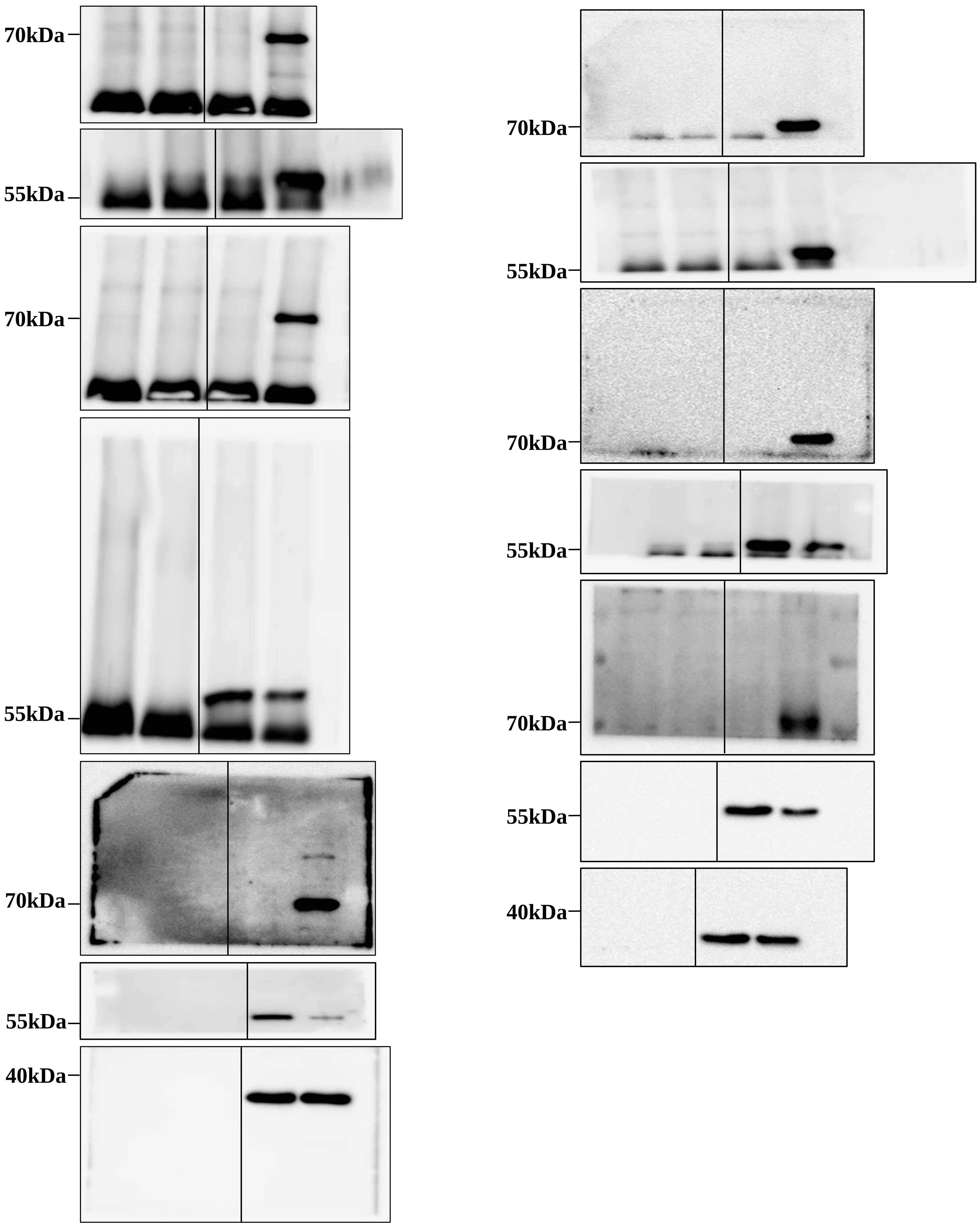
70kDa
55kDa
70kDa
55kDa
70kDa
55kDa
40kDa
70kDa
55kDa
70kDa
55kDa
70kDa
55kDa
40kDa

Fig 5e

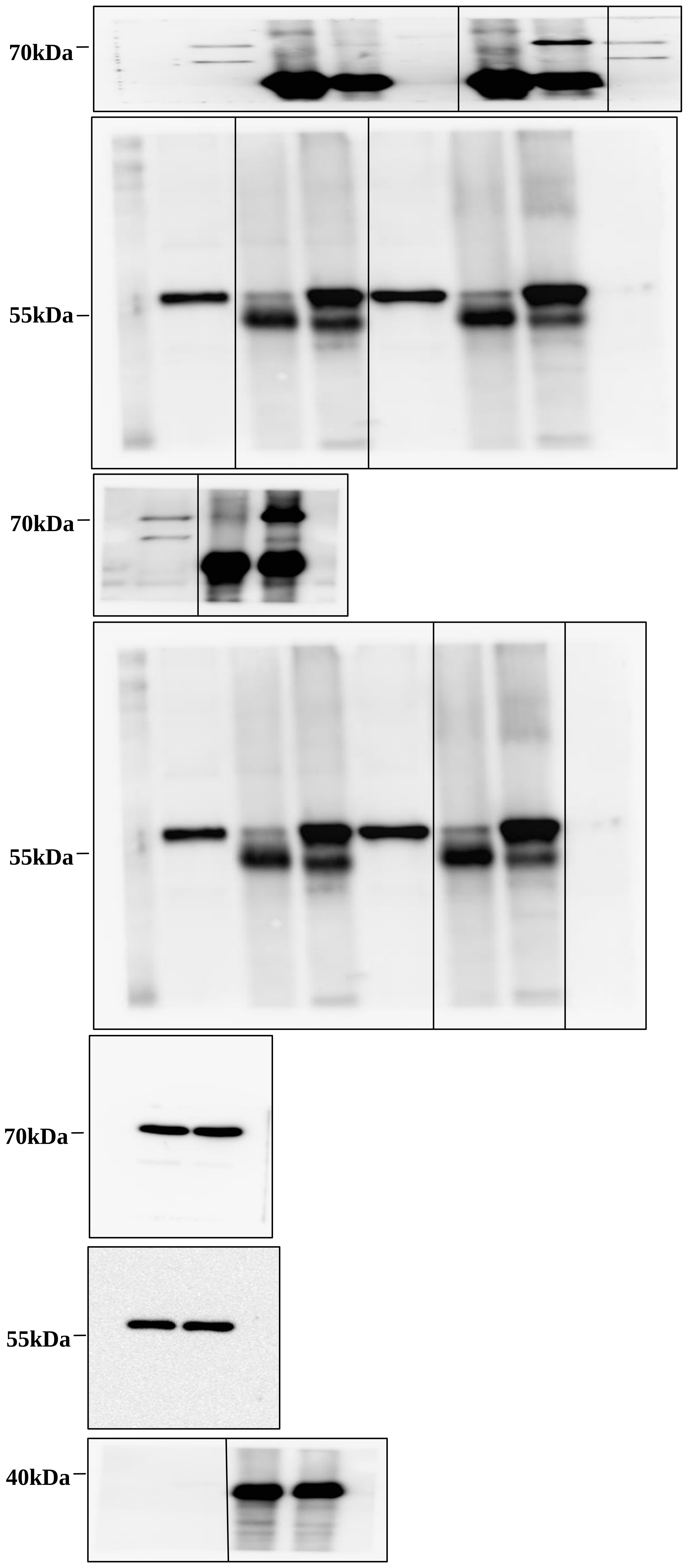

Fig 5f

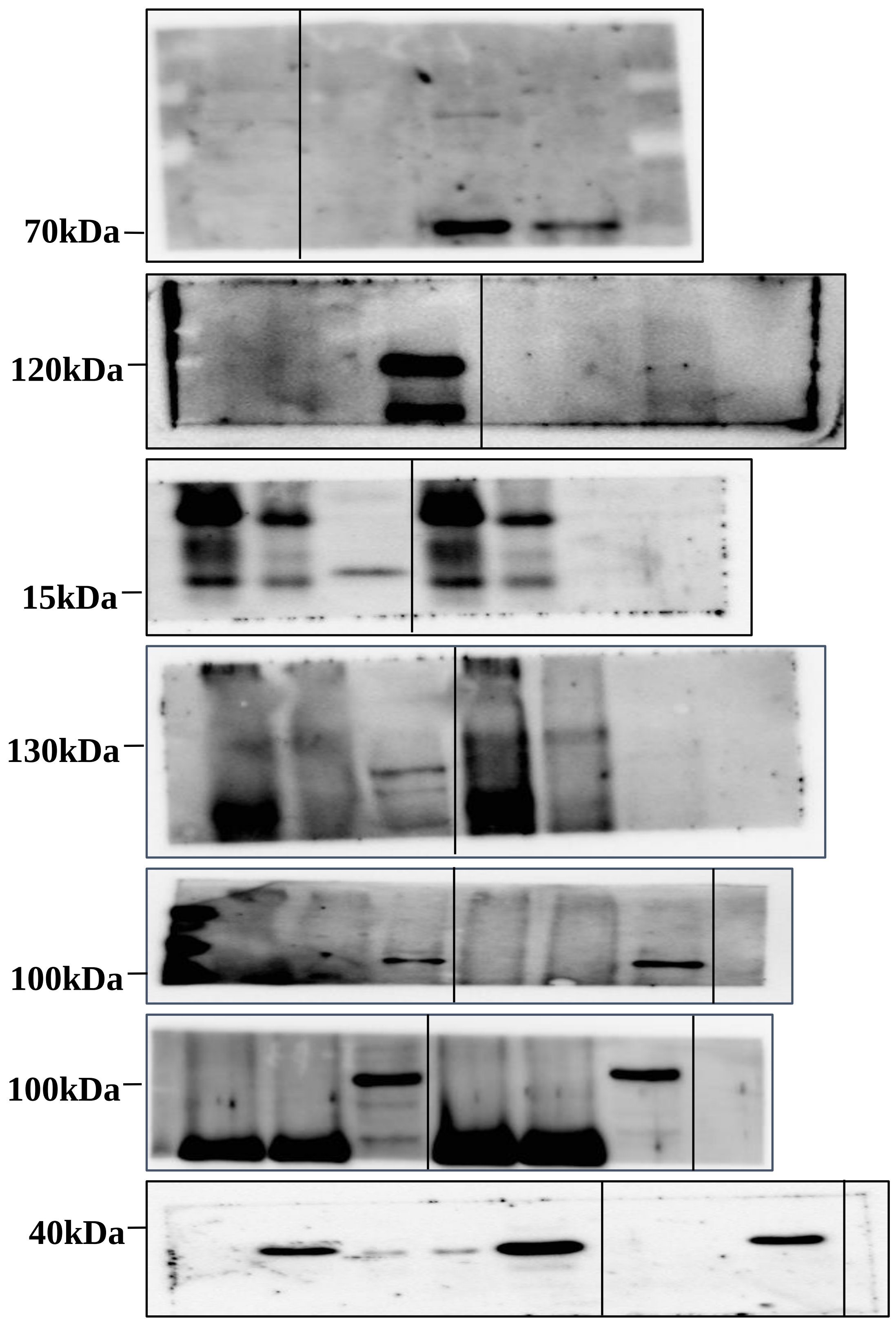

Fig 5g

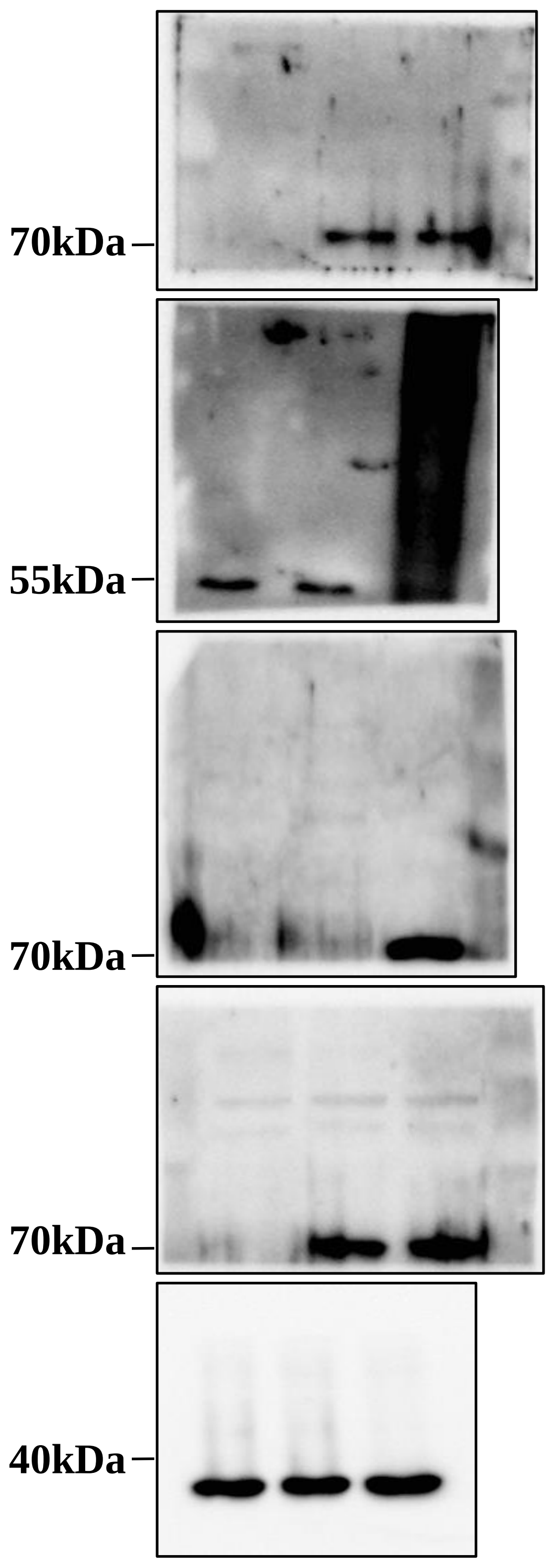

Fig 5h

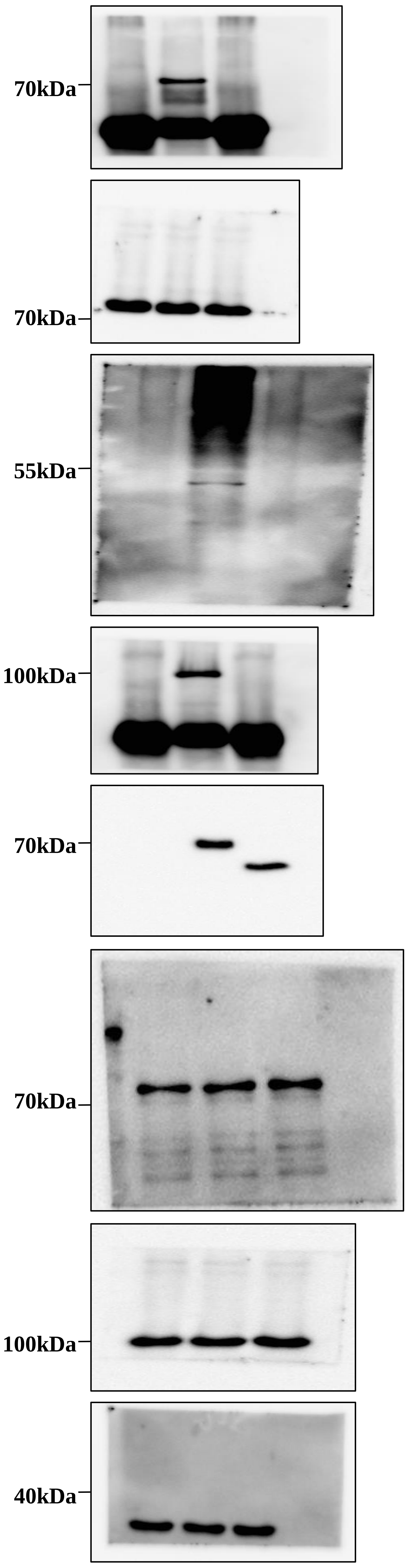

Fig 5i

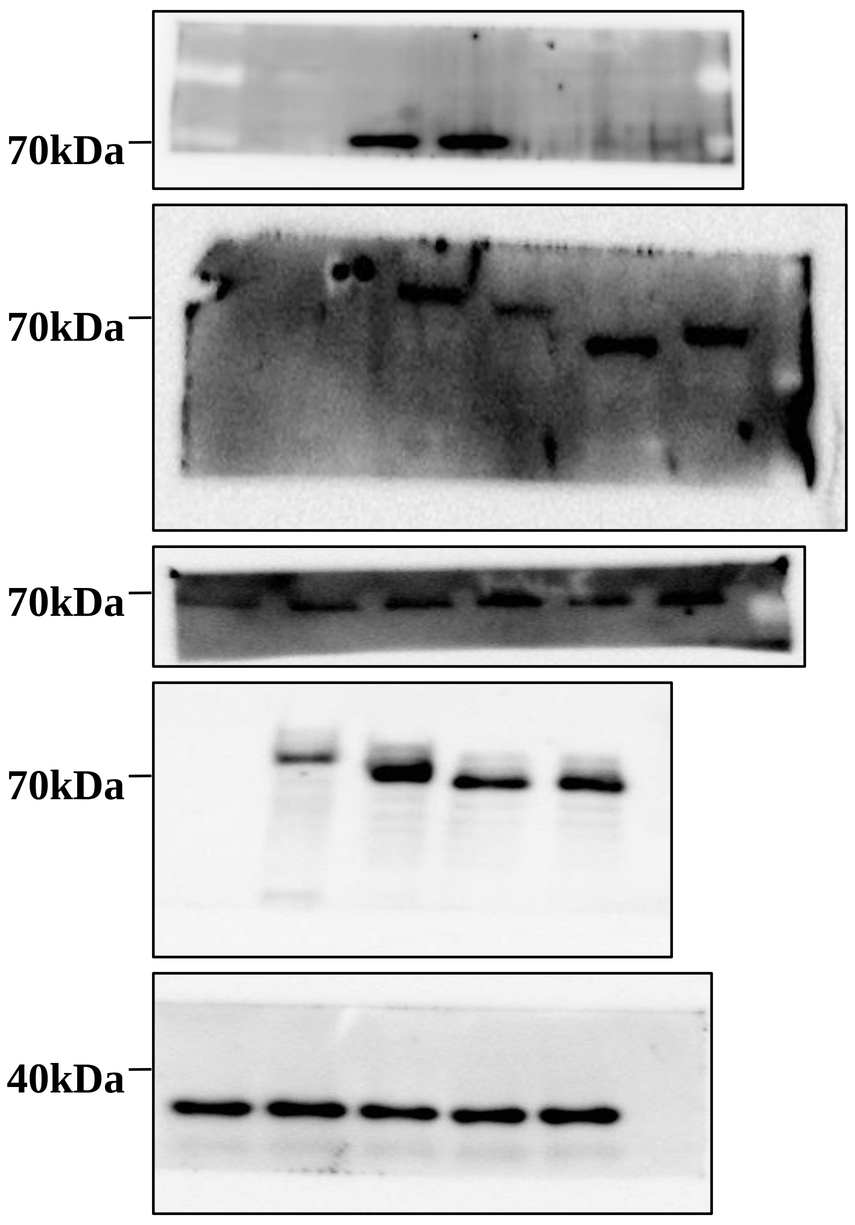

Fig 6a

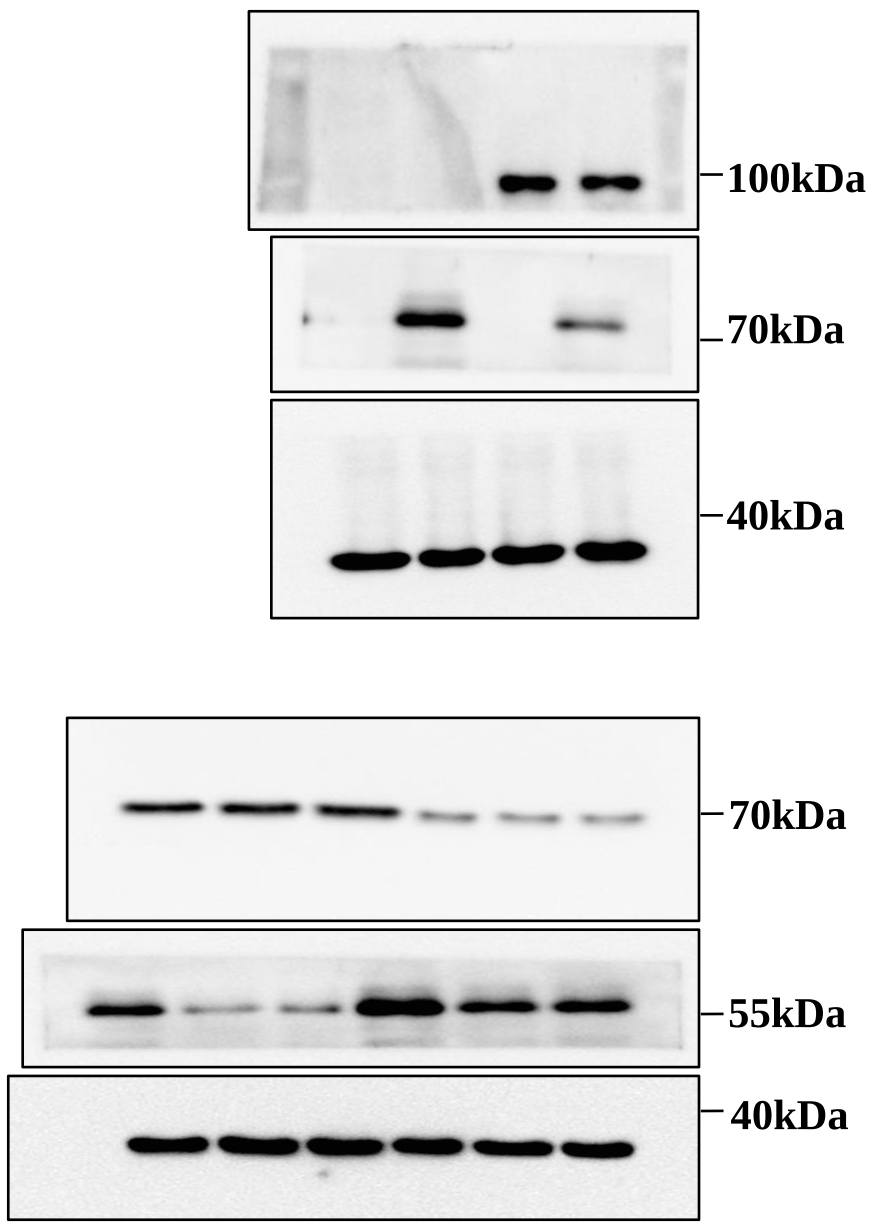

Fig 7e

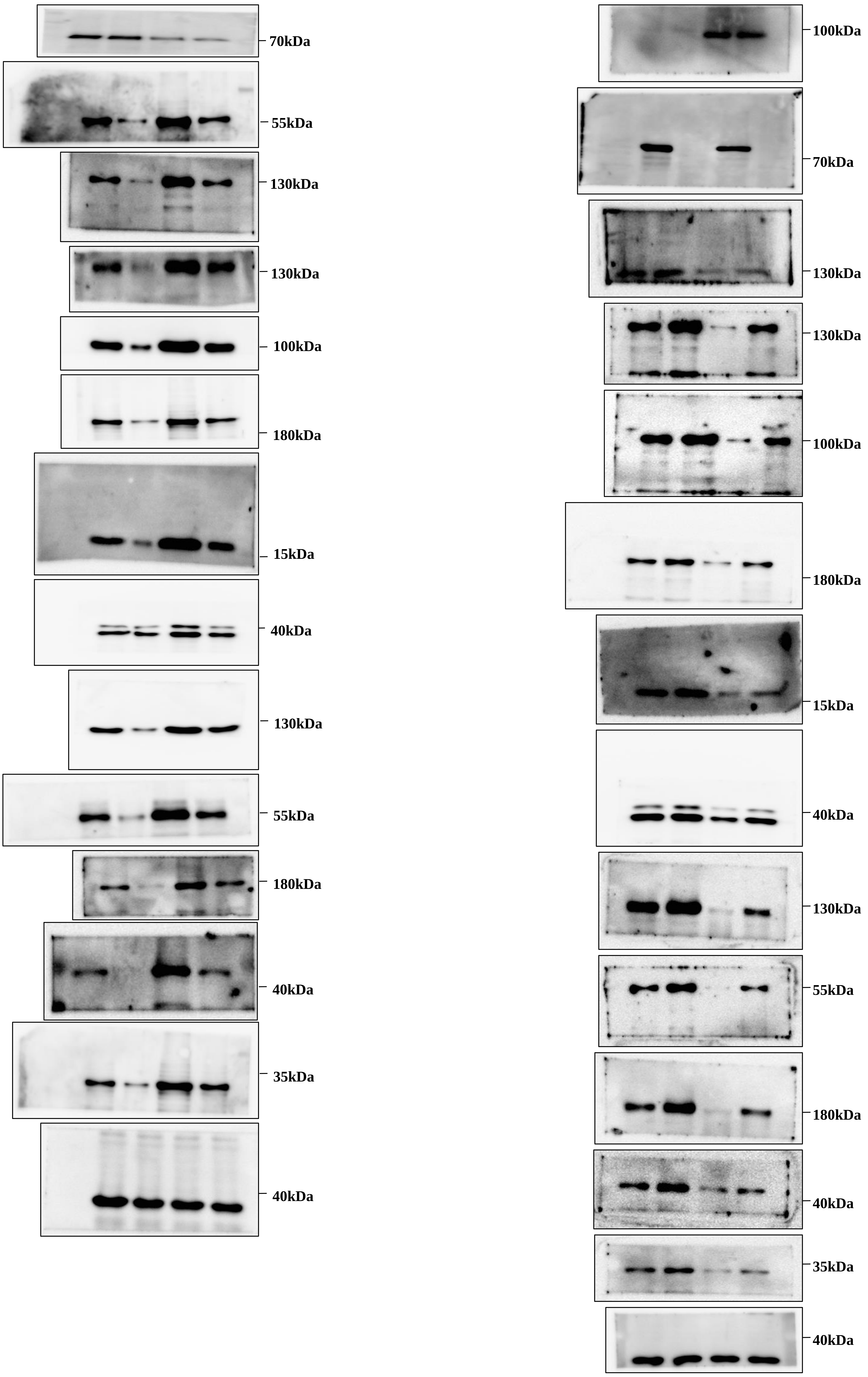
70kDa
55kDa
130kDa
130kDa
100kDa
180kDa
15kDa
40kDa
130kDa
55kDa
180kDa
40kDa
35kDa
40kDa
100kDa
70kDa
130kDa
130kDa
100kDa
180kDa
15kDa
40kDa
130kDa
55kDa
180kDa
40kDa
35kDa
40kDa

Fig S3a

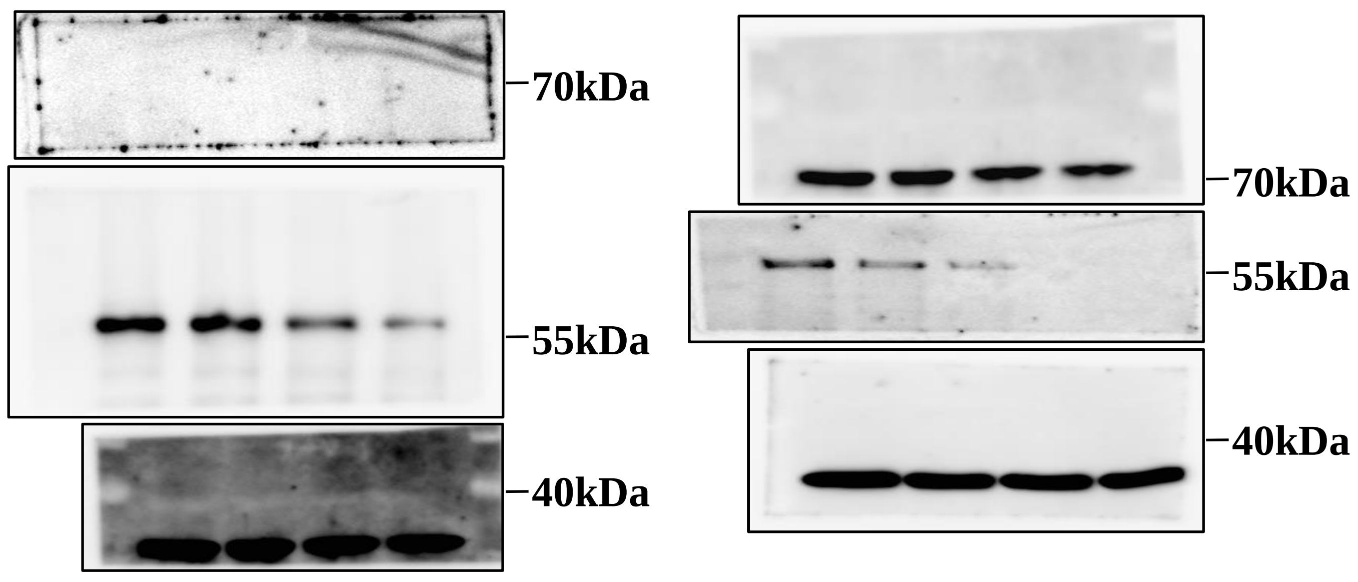

Fig S3b

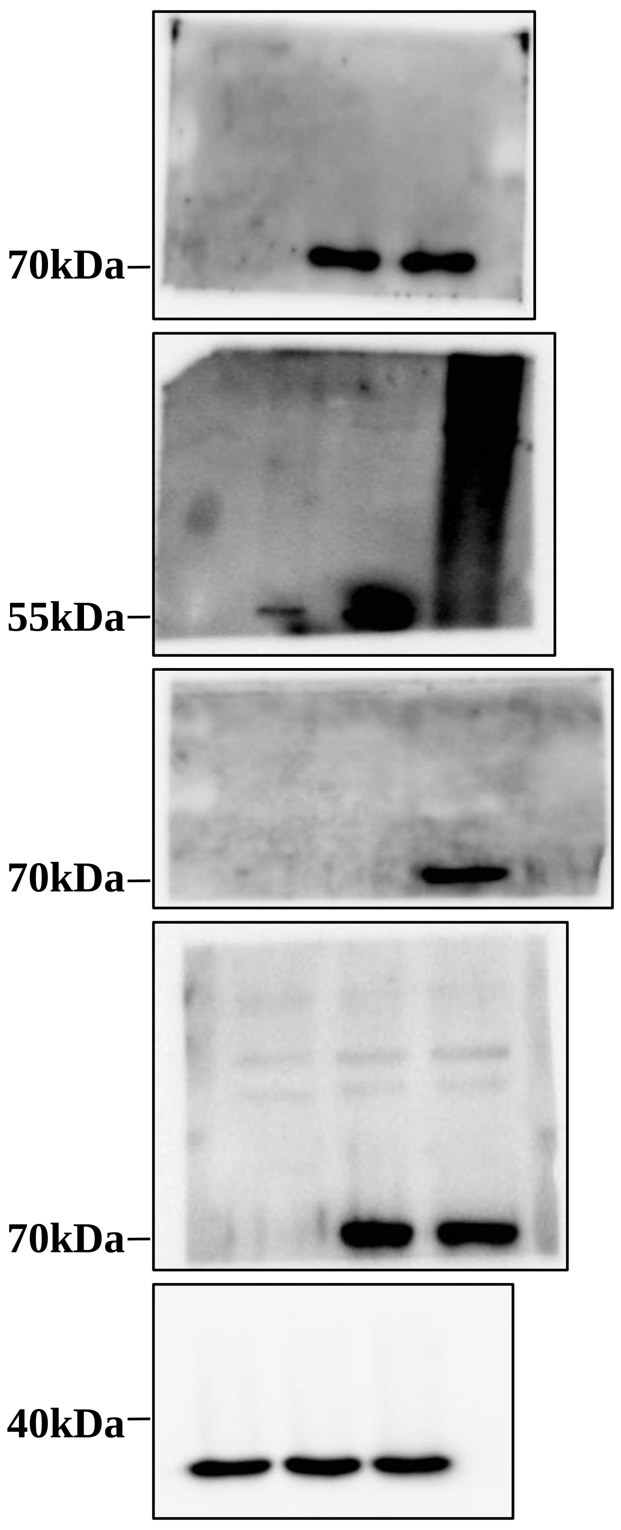

Fig S3c

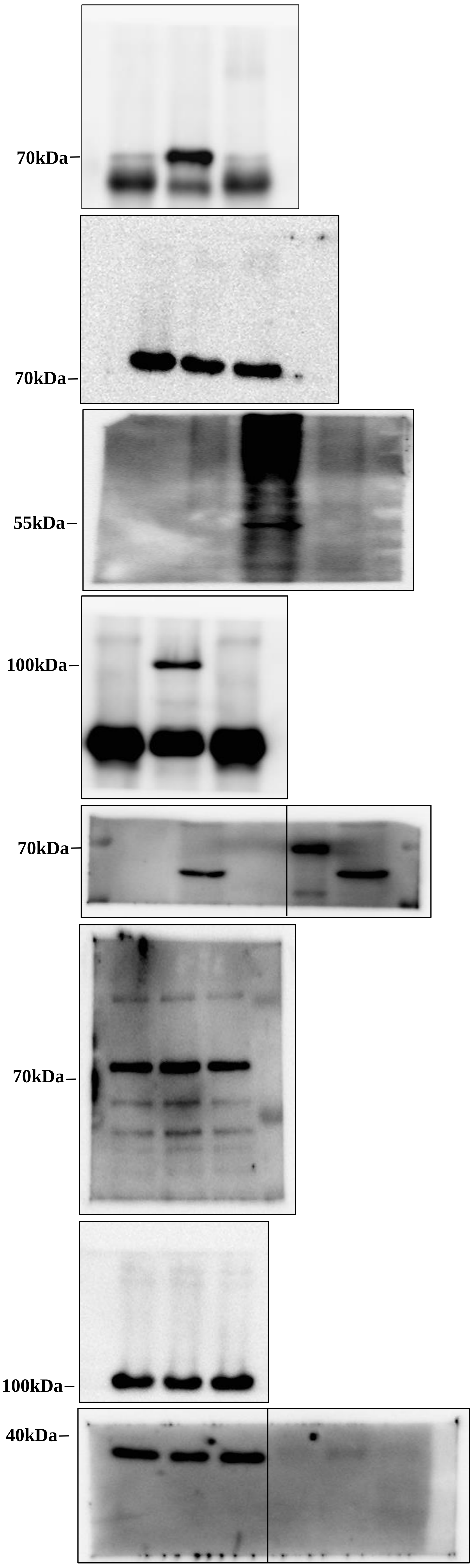

Fig S3d

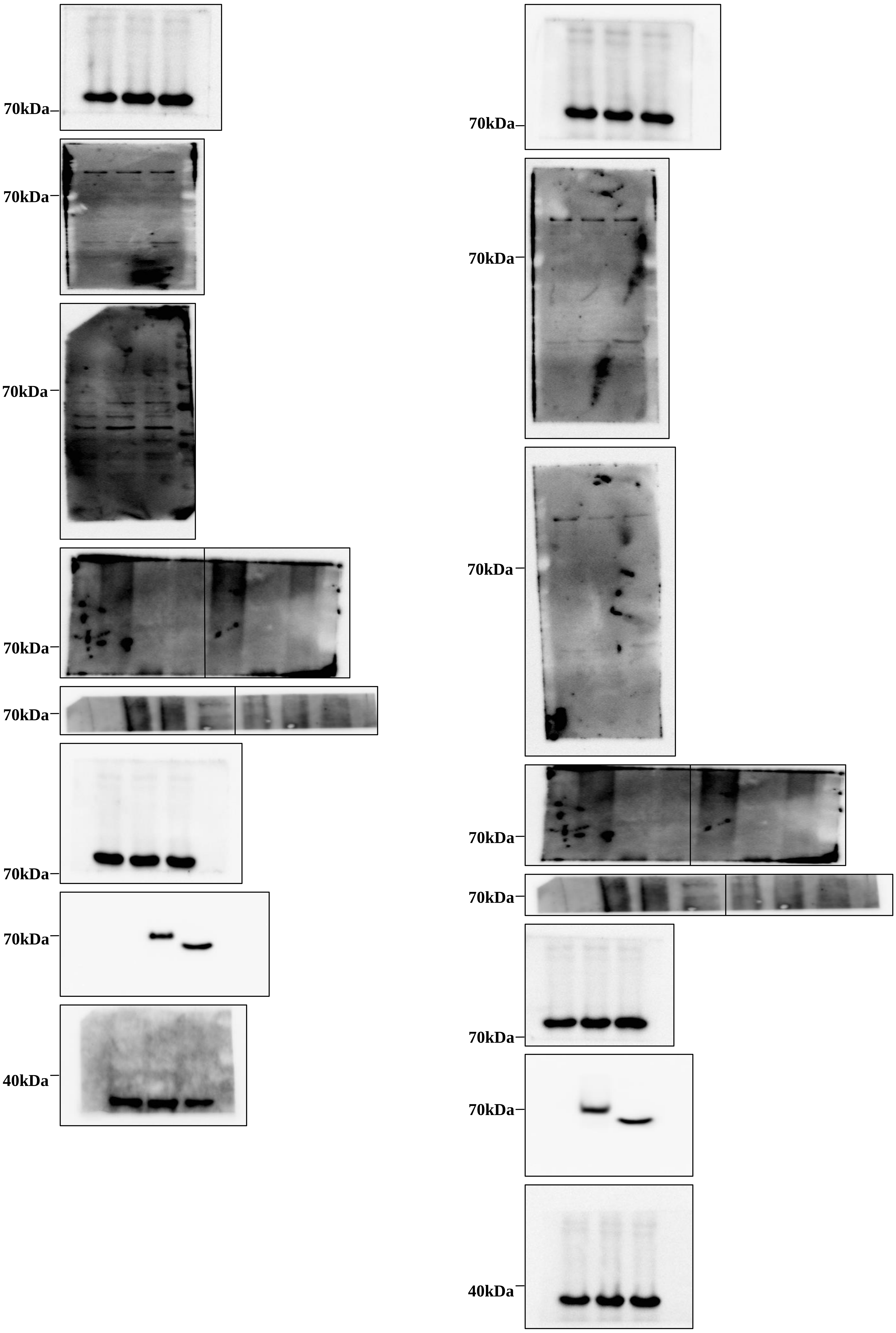

Fig S4a

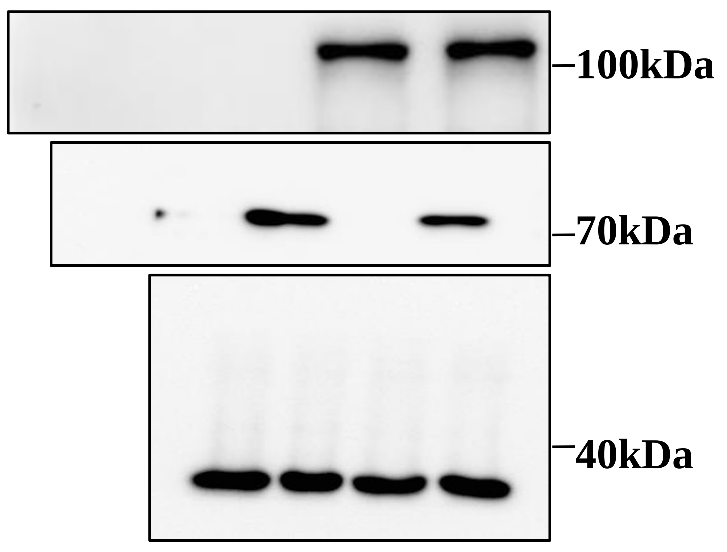

Fig S4f

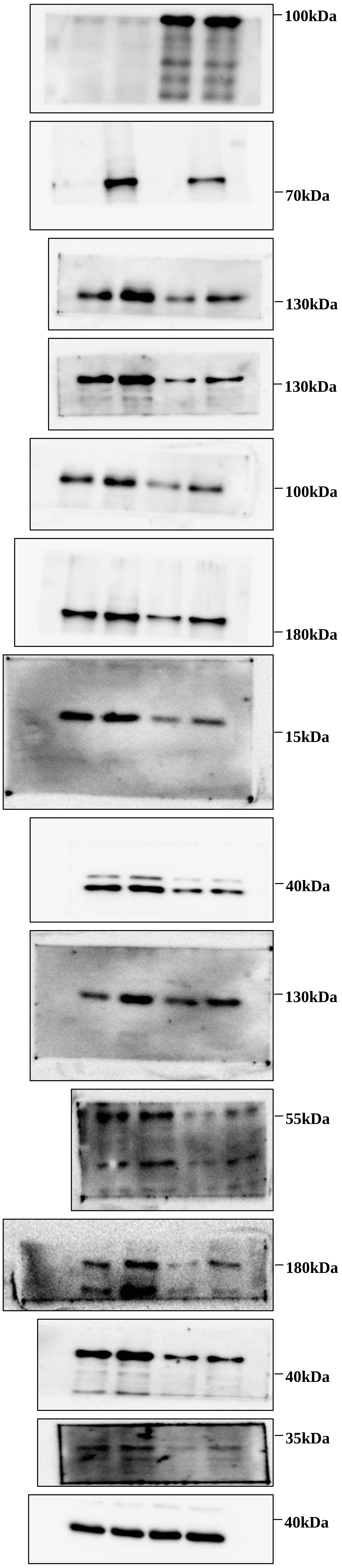
100kDa
70kDa
130kDa
130kDa
100kDa
180kDa
15kDa
40kDa
130kDa
55kDa
180kDa
40kDa
35kDa
40kDa
